# Patterns of gene expression during *Arabidopsis* flower development from the time of initiation to maturation

**DOI:** 10.1186/s12864-015-1699-6

**Published:** 2015-07-01

**Authors:** Patrick T. Ryan, Diarmuid S. Ó’Maoiléidigh, Hajk-Georg Drost, Kamila Kwaśniewska, Alexander Gabel, Ivo Grosse, Emmanuelle Graciet, Marcel Quint, Frank Wellmer

**Affiliations:** Smurfit Institute of Genetics, Trinity College Dublin, Dublin, Ireland; Institute of Computer Science, Martin Luther University Halle–Wittenberg, Halle (Saale), Germany; Department of Biology, National University of Ireland Maynooth, Maynooth, Ireland; Leibniz Institute of Plant Biochemistry, Department of Molecular Signal Processing, Halle (Saale), Germany; Present address: Max Planck Institute for Plant Breeding Research, D-50829 Cologne, Germany

**Keywords:** *Arabidopsis thaliana*, Flower development, Organ specification, Transcriptomics, Temporal gene expression, Paralog, Gene expression atlas

## Abstract

**Background:**

The formation of flowers is one of the main model systems to elucidate the molecular mechanisms that control developmental processes in plants. Although several studies have explored gene expression during flower development in the model plant *Arabidopsis thaliana* on a genome-wide scale, a continuous series of expression data from the earliest floral stages until maturation has been lacking. Here, we used a floral induction system to close this information gap and to generate a reference dataset for stage-specific gene expression during flower formation.

**Results:**

Using a floral induction system, we collected floral buds at 14 different stages from the time of initiation until maturation. Using whole-genome microarray analysis, we identified 7,405 genes that exhibit rapid expression changes during flower development. These genes comprise many known floral regulators and we found that the expression profiles for these regulators match their known expression patterns, thus validating the dataset. We analyzed groups of co-expressed genes for over-represented cellular and developmental functions through Gene Ontology analysis and found that they could be assigned specific patterns of activities, which are in agreement with the progression of flower development. Furthermore, by mapping binding sites of floral organ identity factors onto our dataset, we were able to identify gene groups that are likely predominantly under control of these transcriptional regulators. We further found that the distribution of paralogs among groups of co-expressed genes varies considerably, with genes expressed predominantly at early and intermediate stages of flower development showing the highest proportion of such genes.

**Conclusions:**

Our results highlight and describe the dynamic expression changes undergone by a large number of genes during flower development. They further provide a comprehensive reference dataset for temporal gene expression during flower formation and we demonstrate that it can be used to integrate data from other genomics approaches such as genome-wide localization studies of transcription factor binding sites.

**Electronic supplementary material:**

The online version of this article (doi:10.1186/s12864-015-1699-6) contains supplementary material, which is available to authorized users.

## Background

The formation of flowers is one of the main models for studying the molecular mechanisms underlying the control of plant development. Over the past three decades, a large number of regulatory genes, which control a multitude of different processes during flower morphogenesis, have been identified mainly through a combination of forward and reverse genetics approaches [1–3]. Work in *Arabidopsis thaliana* in particular has led to an understanding of the molecular mechanisms underlying the functions of many of these regulatory genes [4]. Furthermore, it has yielded detailed insights into the regulatory hierarchies among genes that play roles in the control of floral organ formation [5, 6].

With the advent of the genomics era, genetic approaches employed to elucidate the regulation of flower development have been complemented by methods such as global transcript profiling and genome-wide localization studies of transcription factor binding sites. Unfortunately, this work has been hampered in *Arabidopsis* by the fact that flowers of this model plant are small and early-stage floral buds are too minute to be dissected reliably through conventional approaches. Also, *Arabidopsis* flowers are initiated sequentially so that all flowers in an inflorescence are at distinct developmental stages [7]. As a consequence, the collection of sufficient numbers of flowers at particular stages for analysis by genomic technologies is challenging especially for early flower development. To circumvent this problem, a number of approaches have been employed: recently, laser capture microdissection has been used to generate transcriptional profiles of early-stage floral buds [8]. An alternative and largely complementary approach has been the use of floral induction systems, which allow the collection of hundreds of synchronized floral buds from a single plant (see below). These systems have been employed to study both temporal and spatial gene expression during the early stages of flower development [9–14]. Other studies have analyzed gene expression in whole inflorescences of wild-type and mutant plants and in some cases relied on the removal of older (and relatively large) buds that may unduly contribute to RNA preparations from these tissues [15–19]. Moreover, transcript profiling was done with wild-type flowers at individual stages and with distinct floral organ types, but this work has been limited to older flowers, as they can be collected with relative ease [17]. Specific developmental processes such as male-gametophyte/pollen and female gametophyte/ ovule development have also been studied through transcriptomics experiments, providing detailed information for individual cell and tissue types [20–23].

Although *Arabidopsis* flower development has been studied extensively over the past ten years through the genomics approaches described above, a continuous series of gene expression from the time of initiation to maturation has been lacking. Obtaining this information could be highly informative as it would provide a comprehensive view of stage-specific gene expression activities over the entire course of development and would constitute an important component of a gene expression map. Furthermore, such a dataset could be used in analyses, in which, for example, data from transcript profiling and genome-wide localization studies are integrated to obtain a better understanding of the gene network that controls flower formation.

In this study, we employed a floral induction system to close this knowledge gap and to monitor temporal gene expression during flower development from the time of initiation to maturation. We validated the resulting dataset and used it to obtain novel insights into the processes underlying the formation of flowers on a global scale through computational approaches.

## Results and discussion

### Temporal gene expression during flower development

**Fig. 1:**
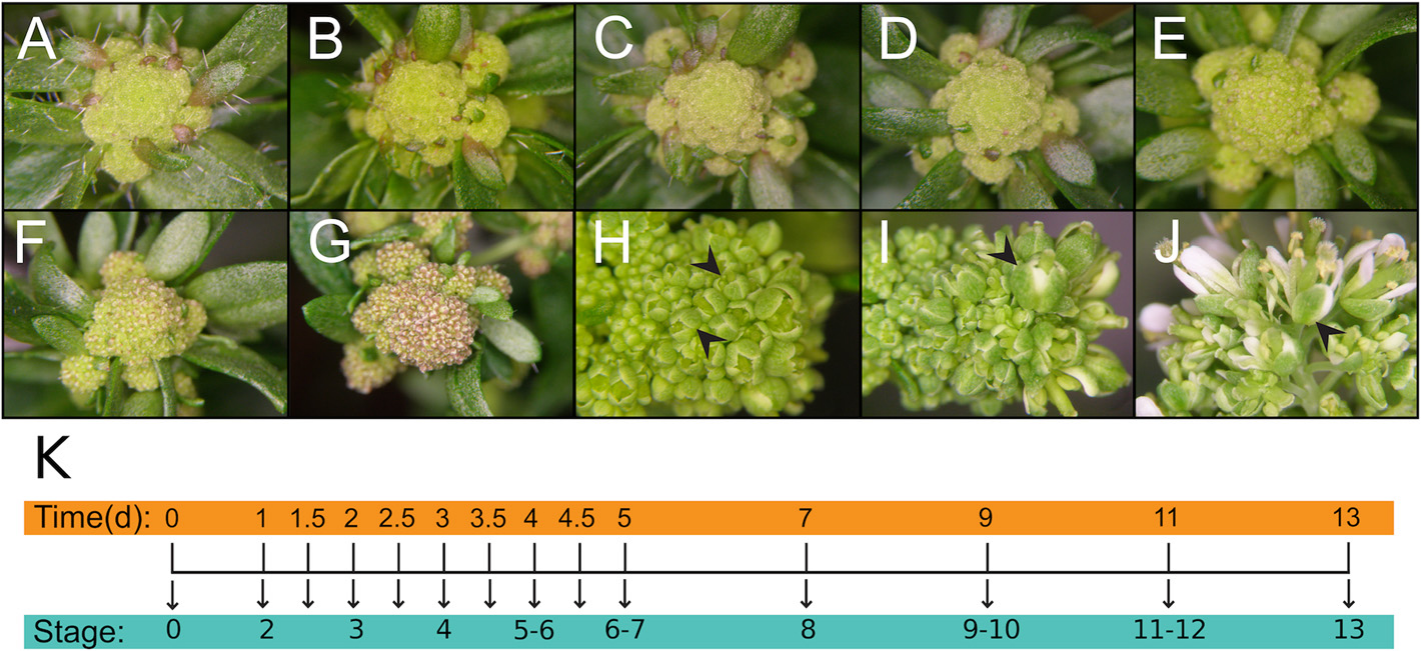
Analysis of temporal gene expression during flower development. **a-j** Inflorescences of AP1_pro_:AP1-GR *ap1-1 cal-1* plants **a** before dexamethasone treatment (0 d time-point) , and **b** 1 d, **c** 2 d, **d** 3 d, **e** 4 d, **f** 5 d, **g** 7 d, **h** 9 d, **i** 11 d, and **j** 13 d after treatment with a solution containing 10 μM dexamethasone. The development of flowers on a given inflorescence was largely synchronous until day 7. For later time-points (**h-j**), flowers were harvested from the tip of the inflorescences (arrowheads) after phenotypic assessment. **k** Experimental set-up used for this study. Floral buds were collected from the inflorescences of AP1_pro_:AP1-GR *ap1-1 cal-1* plants at 14 time-points immediately before and after treatment with a dexamethasone (‘DEX’)-containing solution, which induces flower development by activating the AP1-GR fusion protein. Floral buds from the time of initiation until anthesis (corresponding to stage 13) were sampled

To identify patterns of gene expression during flower development from the time of initiation to maturation (stage 13; stages according to [7]), we employed a previously described floral induction system, which allows the collection of hundreds of floral buds from a single plant [9, 13, 24, 25]. This system is based on the expression of the floral meristem identity factor APETALA1 (AP1) fused to the hormone-binding domain of the rat glucocorticoid receptor (GR) from the *AP1* regulatory region (AP1_pro_) in an *ap1 cauliflower* (*cal*) double-mutant background. *Ap1 cal* plants accumulate inflorescence-like meristems at their shoot apices [26, 27], and activation of the AP1-GR fusion protein in this background through treatment of the plants with the steroid hormone dexamethasone results in the transformation of these meristems into floral primordia, which subsequently develop in a largely synchronized manner. However, at intermediate stages, this synchronization is gradually lost likely due to space constraints [9]. Despite this overall loss of synchronization, we noticed that flowers at the very tip of the inflorescence heads remained fairly synchronized throughout flower development perhaps due to a larger degree of curvature in his area, which may allow floral buds to develop without coming into contact with neighboring flowers. For the gene expression profiling experiments, we therefore collected older floral buds (days 9 to 13 after dexamethasone treatment, corresponding to stages 9-10 to 13, respectively) from this region alone, while younger flowers were harvested more liberally from the inflorescences of AP1_pro_:AP1-GR *ap1 cal* plants (Fig. 1a-j). To obtain expression data for a large number of distinct floral stages, we collected floral buds at 14 different time-points either immediately before (referred to as 0 d time-point) or from 1 to 13 d after the induction of flower development through treatment with dexamethasone (Fig. 1k). Because early flower development is characterized by dramatic changes in morphology [7] and involves a large number of transcriptional regulators that control important processes such as floral patterning and floral organ specification [4], we collected most samples at those stages with intervals in-between time-points ranging from 0.5 to 1 d. At later stages of development, the intervals for sample collection were extended to 2 d (Fig. 1k).

**Fig. 2:**
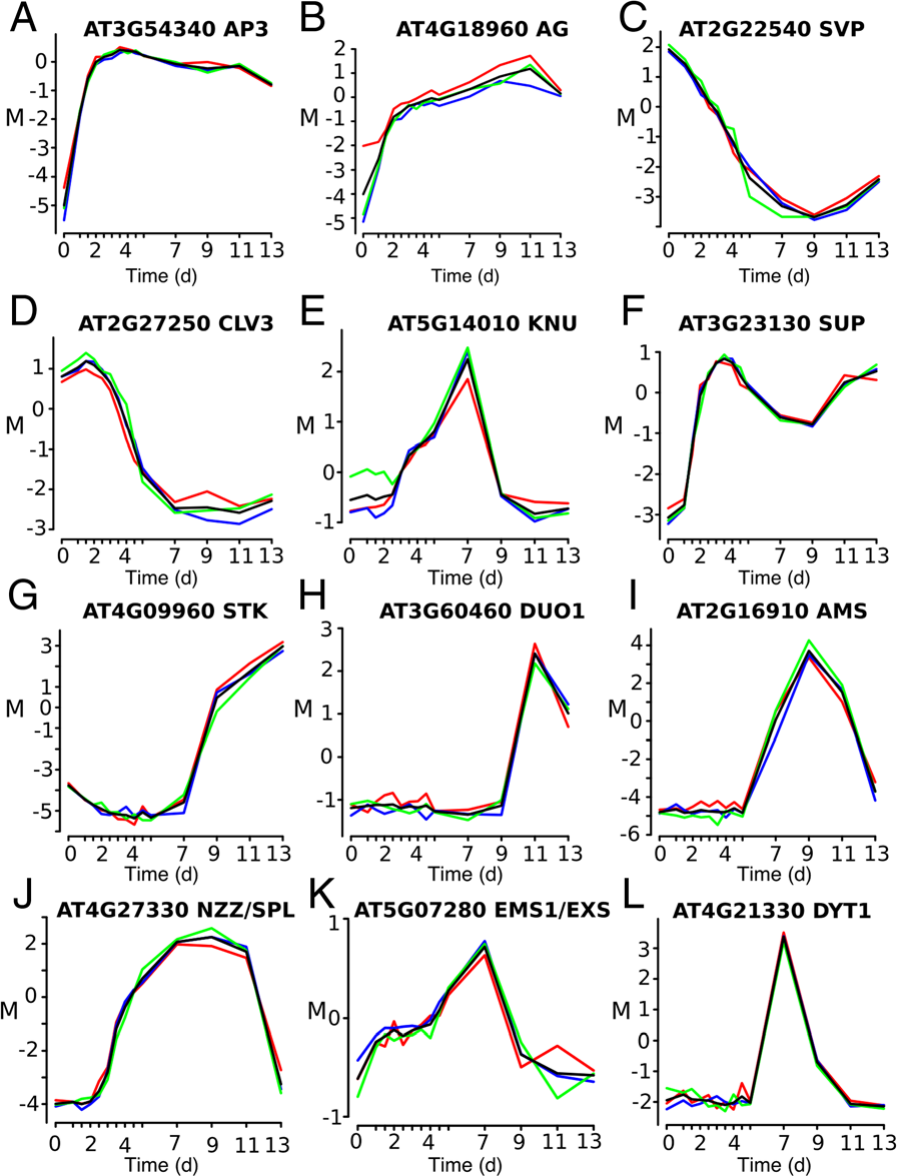
Expression profiles of known floral regulators. **a-l** *M* values (log_2_ (expression in sample/expression in common reference)) for selected floral regulators (as indicated) are shown for all time-points. Red, green and blue lines represent data from three biologically independent sets of samples, black lines the mean values of the replicate experiments. Note the high reproducibility of the expression data across all time-points

For microarray analysis of the tissue samples, we employed a common reference design (e.g., ref. [28]). We then assessed the resulting data for reproducibility and found that the replicates for the individual time-points correlated well (Figure S1 in Additional file 1; see also Fig. 2), implying that the progression of flower development and the tissue collection was highly reproducible over the entire course of the experiment. In order to determine significant expression changes, we applied an F-statistic and searched across the entire dataset for genes with differential expression. We identified ~20,000 genes (i.e., ~75 % of the genes in the *Arabidopsis* genome) that showed differential expression in at least one of fourteen time-points. Because many of these transcriptional changes may be caused by the dramatic alterations in floral size and morphology during the course of development and not by specific gene regulatory events, we next sought to identify genes whose expression changed relatively rapidly. To this end, we compared gene expression between consecutive as well as near-by (within a 2-d time interval) time-points to minimize the effects of morphological alterations and identified 7,405 genes as differentially expressed (Additional file 2). Many of these differentially expressed genes (DEGs) were detected at intermediate (between 5 and 9 d after dexamethasone treatment) and late (between 9 and 13 d) stages of flower development, and overall, a preponderance of gene activation over repression was observed (Table S1 in Additional file 1). Although we found many genes to be repressed immediately after the onset of flower development, this effect was not as pronounced as previously described [9, 29], possibly because of the different floral induction systems and/or different experimental set-ups and data analysis pipelines used.

To validate the results of the microarray experiments, we assessed the expression profiles of genes with known roles in different processes during flower development (Fig. 2 and Figure S2 in Additional file 1) and found that they were in concurrence with their published expression patterns. For example, expression of the floral homeotic genes *APETALA3* (*AP3*) and *AGAMOUS* (*AG*) (Fig. 2a-b) strongly increased in early time-points and then remained high throughout most of flower development in agreement with the activation of these genes at stage 3 and their continued expression in developing floral organs [30, 31]. Down-regulation of the floral repressor *SHORT VEGETATIVE PHASE* (*SVP*) (Fig. 2c) at early floral stages has been described previously and is dependent on AP1 activity [29, 32]. Expression of the stem cell regulator *CLAVATA3* (*CLV3*) was high at early stages and then rapidly decreased in intermediate-stage flowers (Fig. 2d) likely as a consequence of the loss of floral stem cells around stage 6 of development [33]. This termination of floral meristems is at least in part due to the activity of *KNUCKLES* (*KNU*), which we detected to be expressed at intermediate stages (Fig. 2e), in agreement with its known expression pattern at the base of developing carpels and in stamen primordia [34, 35]. Genes with bimodal expression profiles included *SUPERMAN* (*SUP*) (Fig. 2f), which is initially expressed in young floral meristems and at later floral stages during ovule development [36]. Strong up-regulation of the regulator of ovule and seed development *SEEDSTICK* (*STK*) between days 7 and 9 in our experiment (Fig 2g) corresponds to its expression in developing carpels from stage 8 onward [37]. *DUO POLLEN1* (*DUO1*), a regulator of male germline development, was found to be expressed in late flower development (Fig. 2h) in agreement with its specific expression in pollen [38]. *ABORTED MICROSPORES* (*AMS*), which encodes a master regulator of pollen wall formation, was strongly expressed at intermediate stages and reached a maximum around stages 9-10 (9 d after dexamethasone treatment) (Fig. 2i) as previously described [39]. Genes such as *NOZZLE/SPOROCYTELESS* (*NZZ/SPL*) (Fig. 2j), *EXTRA MICROSPOROCYTES1*/ *EXTRA SPOROGENOUS CELLS* (*EMS1/EXS*) (Fig. 2k), and *DYSFUNCTIONAL TAPETUM1* (*DYT1*) (Fig. 2l) were expressed during intermediate stages in agreement with their function in early anther development [40–44]. Activation of *NZZ/SPL* was detected in our experiment around stage 5 and thus earlier than what has been reported previously (i.e. stage 6; [45]). This difference might stem from initially low mRNA levels, which might hamper a reliable detection in *in situ* hybridization or reporter gene essays.

We also compared our dataset to those from several previous studies in which temporal [8–10, 14] and spatial [11, 16] gene expression during flower development had been analyzed either in early or in late-stage flowers using different floral induction systems, laser capture microdissection of wild-type flowers, or through a comparison of the gene expression profiles of inflorescences of floral mutants and of the wild type, respectively. For each pair-wise comparison, we found a significant overlap between the datasets and the one described in this study (Table S2 in Additional file 1 and Additional file 3), further validating the results of our time-course experiment.

### Distribution of functional terms among groups of co-expressed genes

**Fig. 3:**
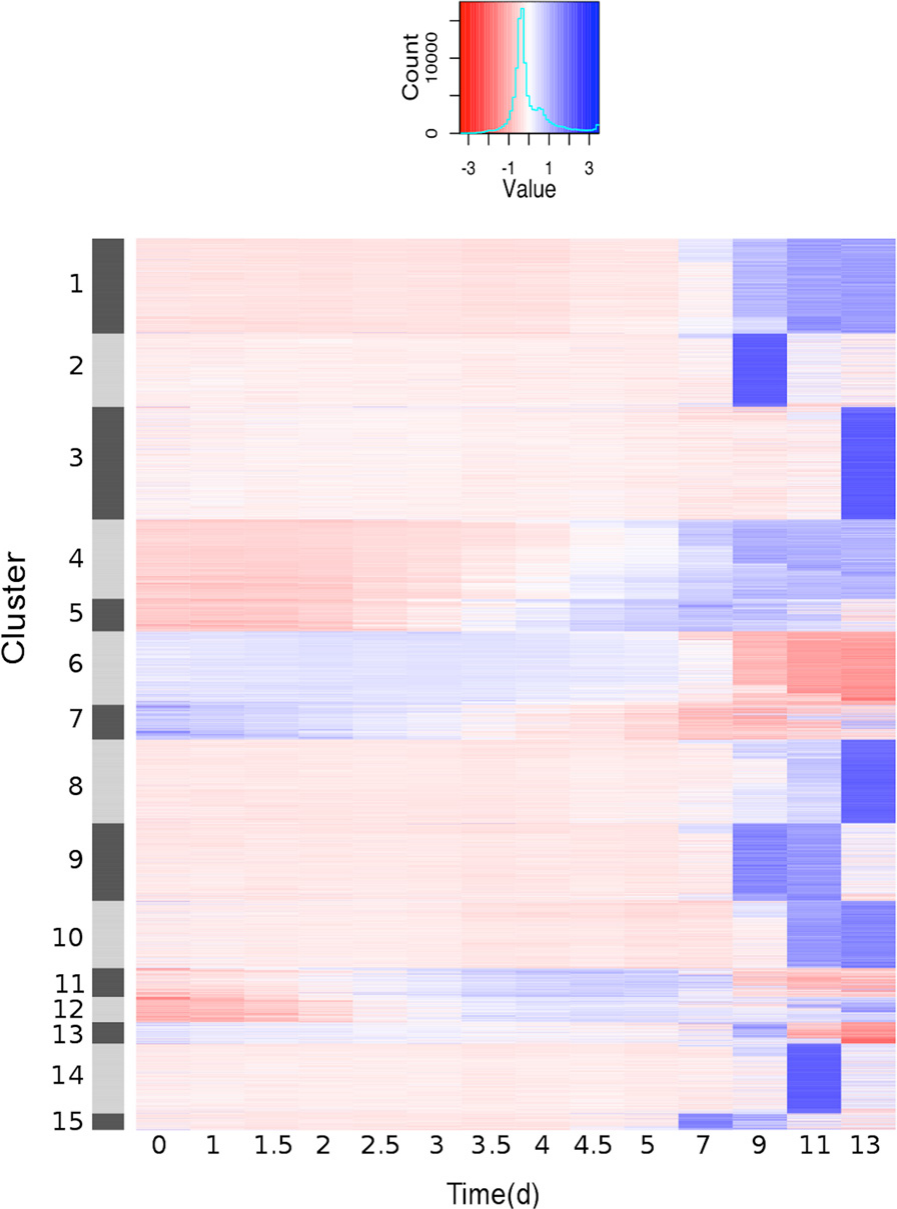
Genes showing differential expression during flower development. Groups of co-expressed genes were identified among 7,405 differentially expressed genes detected in the time-course experiment. The heat map shows the results of *k*-means clustering (*k* = 15) used to group genes based on the similarity of their *z*-scores (color-coded as per diagram at the top). For a different representation of the individual clusters, see Figure S3

Because functionally related genes are often co-expressed during development, we used a *k*-means algorithm to group the DEGs into 15 clusters with distinct gene expression profiles (Fig. 3 and Figure S3 in Additional file 1). Figure 3 shows that the majority of DEGs are predominantly expressed at or after the 9-d time-point. Notable exceptions include genes in clusters 5, 11 and 15, which are up-regulated during early flower development and are repressed at intermediate to late stages. Also, clusters 6 and 7 contain genes that are expressed at the earliest floral stages and are subsequently down-regulated. Genes assigned to clusters 4 and 12 are activated during early flower development when organ primordia are initiated and remain expressed until flowers have reached maturity, suggesting that many of them might play roles during the course of floral organ morphogenesis.

**Fig. 4:**
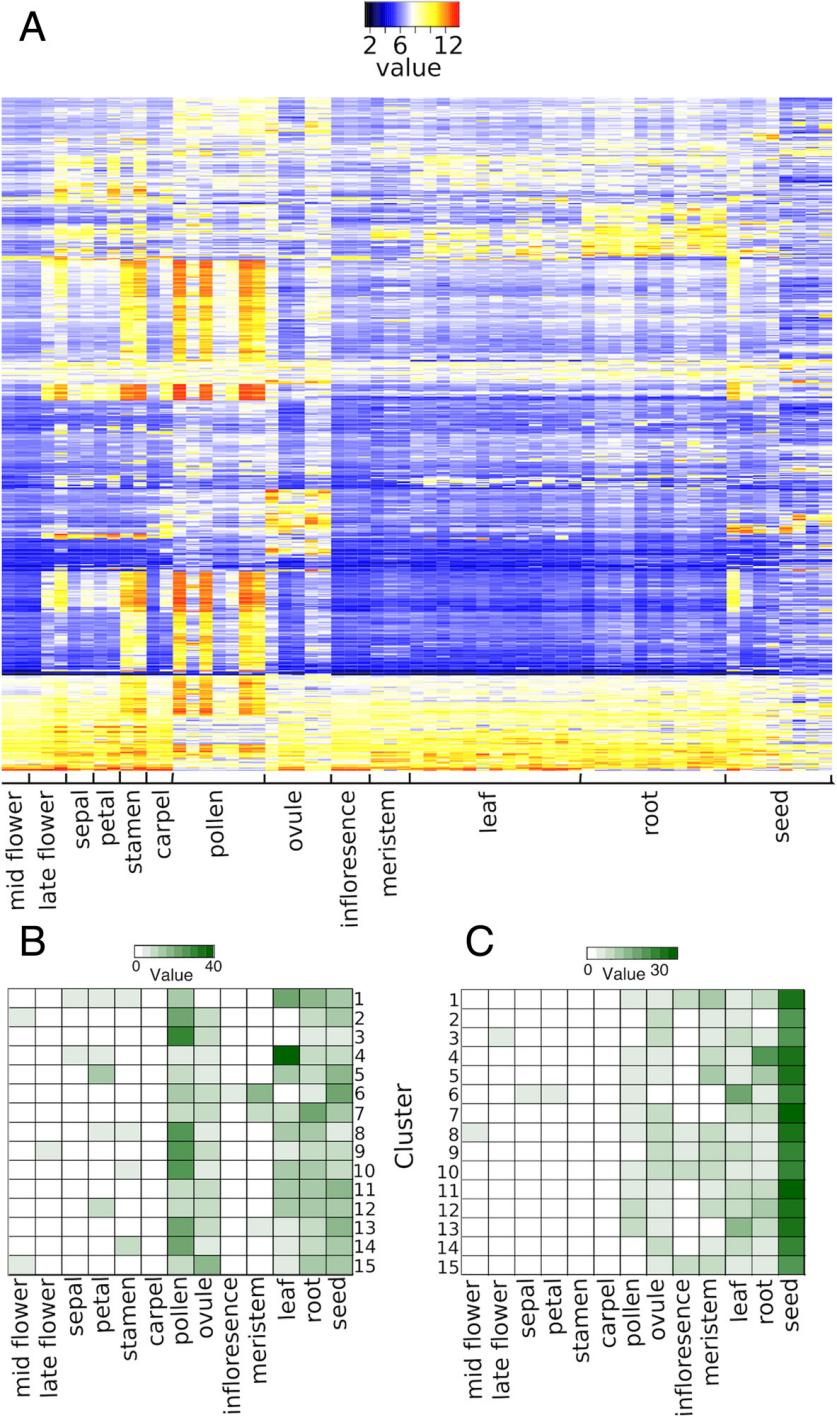
Mapping groups of co-expressed genes onto an *Arabidopsis* gene expression atlas. **a** Expression data for an *Arabidopsis* gene expression atlas were obtained for genes assigned to each of the 15 *k*-means clusters and hierarchical clustering was performed. Results for cluster 3 are shown as an example. Individual tissue and organ samples of the gene expression atlas (shown in full in Additional file 4) were grouped together as indicated. Note a preponderance of expression in stamen and pollen samples. **b** and **c** The number of genes in each cluster with **b** maximum and **c** minimum expression in each of the tissue samples (as indicated) is shown

To obtain insights into the functions of the genes assigned to each of the clusters and to further validate the microarray data, we mapped the groups of co-expressed genes onto an *Arabidopsis* gene expression atlas we had generated previously [13] based on published data (Fig. 4a and Additional file 4). We then determined the percentage of genes with maximum (Fig. 4b) and, for comparison, minimum (Fig. 4c) expression in different groups of related tissue samples. For some of the clusters, this analysis allowed predictions of the predominant location of gene expression. For example, a high percentage of genes with maximum expression in pollen was identified in clusters 2, 3, 8-10, and 13-14. Genes assigned to these clusters were predominantly expressed from or after the 9-d time-point and thus at stages when pollen formation occurs [46]. Clusters 6, 7, and 13 contained the highest proportion of genes with maximum expression in meristems, in agreement with the observation that genes in these clusters are strongly expressed during the earliest floral stages, but are repressed towards more intermediate stages when meristematic activity in flowers ceases. The highest percentage of genes with maximum expression in ovules was found in cluster 15, which contains relatively few genes that are strongly expressed around the 7 and 9-day time-points (corresponding to floral stages 8-10; Fig. 1a) and thus at the time when ovule development commences [47].

**Fig. 5:**
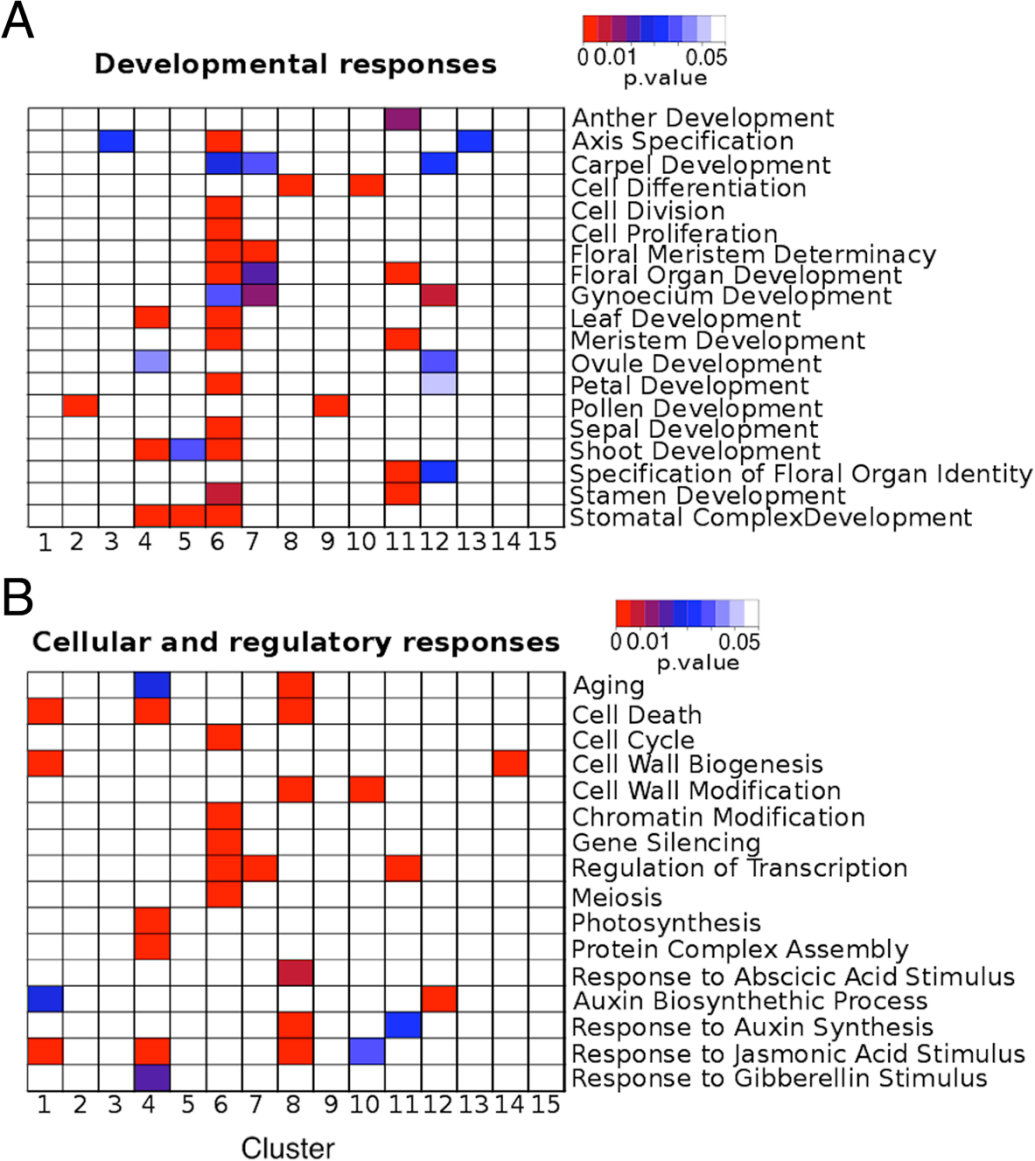
Gene Ontology terms enriched in the dataset. Adjusted *p-*values for selected GO terms related to **a** developmental functions and **b** cellular and regulatory processes are indicated for each cluster through color-coding (see bars at the top right for colors used). For a full list of GO terms enriched in the dataset, see Additional file 5

We also subjected the groups of co-expressed genes to a Gene Ontology (GO) analysis to identify functionally related genes that are significantly enriched (adjusted *p*-value < 0.05) in the individual clusters (Figure S4 and Additional file 5). GO terms directly associated with flower formation (e.g., ‘Specification of Floral Organ Identity’ and terms related to the development of the different floral organ types) and/or floral meristem development (including the terms ‘Cell Proliferation’ and ‘Cell Division’) were found to be enriched, in particular, in clusters 6 and 7, as well as in clusters 11 and 12 (Fig. 5a). As described above, these clusters contain genes that are repressed at early to mid-stages (clusters 6 and 7) or are activated during early flower development (clusters 11 and 12) and remain expressed at least until the end of the intermediate phase of flower development. In agreement with the over-representation of flower-related GO terms in these clusters, they contain many of the regulatory genes (which are also typically associated with the GO term ‘Regulation of Transcription’; see Fig. 5b) known to be involved in controlling the early phase of flower development (Additional file 2). Genes associated with the term ‘Pollen Development’ were enriched in clusters 2 and 9, which contain genes with maximal expression around day 9 of the experiment and hence at a time (corresponding to floral stages 9-10; Fig. 1a) when the microspore mother cells appear and meiosis takes place [46]. Genes involved in cell differentiation were enriched in clusters 8 and 10, which contain genes with predominant expression at late stages of flower development (stages 11-13). Many of these genes exhibit maximum expression in pollen (Fig. 4b) and thus, may be involved to a large extent in the differentiation of microspores into pollen grains. Genes involved in the response to different phytohormones such as jasmonic acid, auxin, and abscisic acid were detected as enriched predominantly in cluster 8, in agreement with the known roles of these hormones in various processes during late-stage flower development, which include stamen and pollen formation as well as the maturation of petals [48]. In contrast, genes involved in the response to gibberellin were over-represented in cluster 4, which contains genes that are induced at the end of the early phase of flower development and remain active until floral maturity has been reached. In agreement with this observation, it has been shown that gibberellins are required for proper floral organ growth and elongation [49]. In sum, the results of these analyses allowed us to attribute specific functions to the individual clusters that together account for many of the processes known to occur during flower development.

### Distribution of target genes of floral organ identity factors

Floral organ identity factors are necessary and sufficient for the specification and development of the different types of floral organs [5, 6]. They act in a combinatorial manner as predicted by the well-supported (A)BCE model of floral organ identity specification [50–52]. Insights into the functions of these master regulators, which (with the exception of APETALA2) all belong to the family of MADS-domain proteins and are components of higher-order regulatory protein complexes [53], have been obtained in recent years through a combination of genome-wide localization studies and gene perturbation experiments [5, 6]. This work has resulted in the identification of some of their direct target genes and of the cellular and developmental processes they control. Furthermore, it has been shown that the floral organ identity factors bind to many of the same sites in the *Arabidopsis* genome [13] and that their global binding patterns undergo changes as flower development progresses, at least in part as a consequence of stage-specific alterations in chromatin accessibility [14]. Also, the majority of genes bound by these transcription factors at early floral stages do not respond transcriptionally when the activities of the floral homeotic genes are perturbed [12, 13]. While the molecular mechanisms underlying these observations are currently not well understood, it is clear that from binding data alone it is difficult to identify their *bona fide* target genes.

**Fig. 6:**
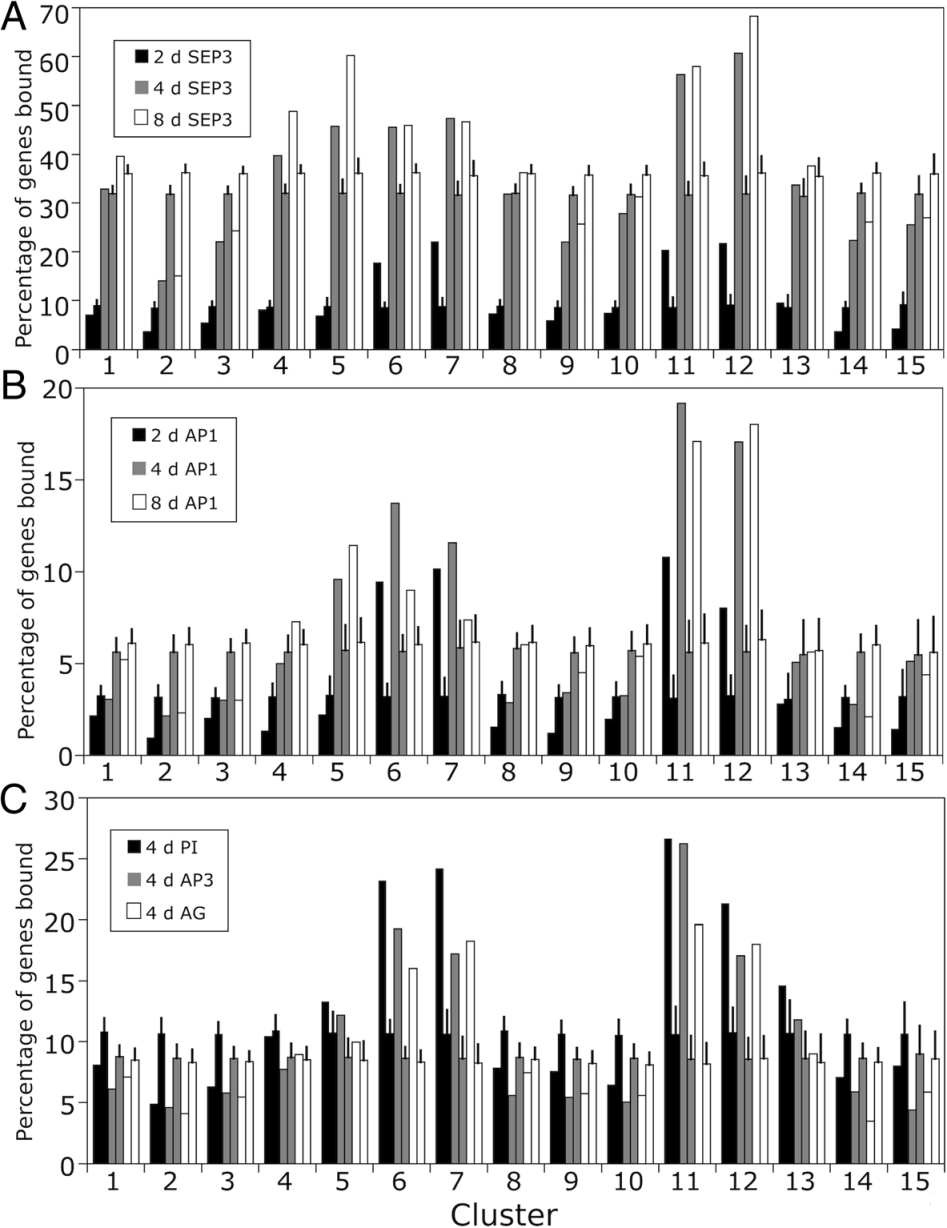
Distribution of genes with binding sites for floral organ identity factors. The percentage of genes in each cluster bound by **a** SEP3, **b** AP1, and **c** AP3, PI, and AG, respectively, is shown. For **a** and **b**, binding data for SEP3 and AP1, respectively, at three different time points after AP1-GR activation were used for analysis: 2 d (black bars), 4 d (gray bars), and 8 d (white bars). For **c**, binding data for AP3 (black bars), PI (gray bars), and AG (white bars) 4 d after AP1-GR activation were used. In all panels, bars without error bars show the results of the comparisons between binding data for the individual transcription factors and the clusters of co-expressed genes, while bars with error bars show the mean percentage of genes bound by a given floral homeotic transcription factor at the indicated time-point in equally sized groups of genes randomly selected from the dataset of 7,405 DEGs. Error bars indicate one standard deviation calculated based on the results of 100 iterations

To test whether we could find evidence for the differential expression of genes that are bound by the floral organ identity factors, we projected the global binding patterns of AP1, SEPALLATA3 (SEP3), AP3, PISTILLATA (PI), and AG onto the dataset from the flowering time-course experiment (Additional file 6). Specifically, we identified the percentage of genes in each of the 15 clusters of co-expressed genes that contain binding sites for these transcription factors in their putative regulatory regions (from 3 kb upstream to 1 kb downstream of the transcribed region of a gene). While binding data for AP3, PI and AG are currently available only for ~ stage 4 flowers [12, 13], for AP1 and SEP3, binding data for three distinct stages (2, 5-6, and 7-8) have been generated [14]. Largely independent of the transcription factor under study, we found the highest degree of binding site enrichment in clusters 6, 7, 11, and 12 (Fig. 6). Cluster 5 also showed a significant enrichment for genes with binding sites, but only for SEP3 and AP1, and not at the earliest (stage 2) time-point. The genes assigned to these different clusters have in common that their transcription changes at the time or shortly after the expression of the floral organ identity genes commences around stage 3. Furthermore, they contain many genes associated with the specification of floral organ identity, as well as the regulation of floral organ development and meristem determinacy (Fig. 5) and thus processes that are known to be under control of the floral organ identity factors [5, 6]. Hence, genes in these clusters containing binding sites for the MADS-domain proteins are good candidates for target genes. In fact, they do contain many of the genes known to act directly downstream of these floral regulators (Additional file 6). However, one caveat of this analysis is that the floral organ identity factors appear to have largely distinct sets of target genes despite their overlapping binding patterns [5]. Therefore, while genes that are differentially expressed during early flower development and that contain binding sites for MADS-domain proteins are likely under control of floral organ identity factors, the exact regulatory complex that might be active in the regulation of a given gene cannot be readily deduced without additional data from floral organ identity gene-specific perturbation experiments.

In addition to clusters with binding site enrichments, we also found clusters that are significantly depleted for binding sites of the floral organ identity factors. These included especially clusters 2, 3, and 14, which contain genes with predominant expression in the time-course experiment at 9, 13, and 11 d, respectively (Fig. 3). As described above, these clusters comprise in all probability many genes involved in microsporogenesis and pollen development, a process that can progress without the direct involvement of the floral organ identity factors [45]. Taken together, this analysis shows that the results of our transcriptomics study can be used as a reference to integrate different genome-wide datasets and to identify candidates for transcription factor target genes.

### Distribution of paralogs within groups of co-expressed genes

**Fig. 7:**
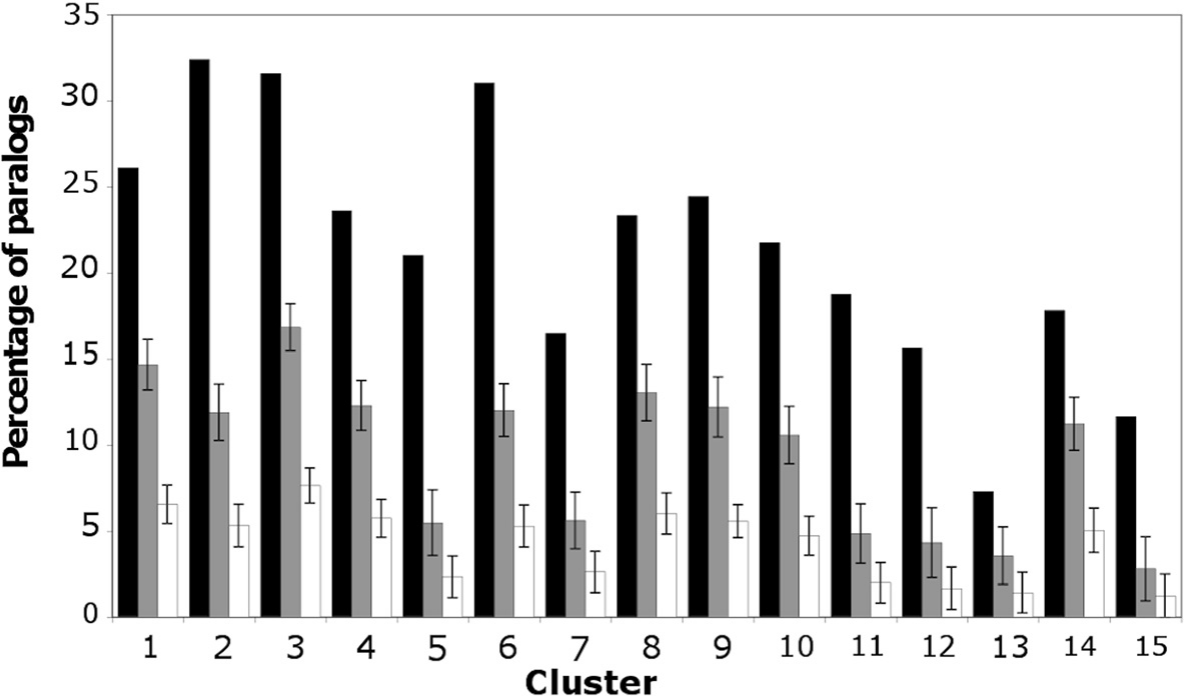
Distribution of paralogs in groups of co-expressed genes. The percentage of paralogs in each cluster of co-expressed genes (black bars) was determined as described in Methods. To identify clusters with a significant enrichment of paralogous genes, the mean percentage of paralogs was determined in equally sized groups of genes randomly selected from the dataset of 7,405 DEGs (gray bars) and from the *Arabidopsis* genome (white bars), respectively. Error bars indicate one standard deviation calculated based on the results of 100 iterations

In plants, duplicated genes that are retained in a genome are often functionally redundant, although sub- or neofunctionalization may lead to paralogous genes that have only partially overlapping activities or that are employed in entirely different developmental processes, respectively [54]. Shared activities of paralogous genes typically go along with overlapping expression patterns. Therefore, one would expect to find in the clusters of co-expressed genes that paralogs are enriched relative to their genome-wide distribution. In fact, it has been shown previously that paralogous genes are over-represented in some but not all groups of genes with predominant expression at certain stages of early flower development [9]. To test whether this unequal distribution of paralogs extends to intermediate or late stages of flower development, we determined paralogs in each of the 15 clusters described in Fig. 3 (for paralogs identified in the clusters, see Additional file 7). As expected, we found that the percentage of paralogs was significantly (i.e., beyond three standard deviations) increased in all clusters relative to their genome-wide distribution and to a lesser extent (and with the exception of cluster 13) relative to their distribution within the 7,405 DEGs as well (Fig. 7). Notably, the enrichment of paralogs within the clusters varied considerably, with clusters 5, 11-12, and 15 having the highest enrichment values (Table S3 in Additional file 1). In agreement with the idea that genes involved in floral organ development exhibit an increased level of genetic redundancy [9], the genes in these clusters have in common that they are activated during early or intermediate (cluster 15) stages of flower development and many of them have known functions in floral organ morphogenesis and in the control of floral meristem determinacy (Fig. 5). In sum, our results further highlight the varying degree to which paralogous genes contribute to different processes during flower development. Whether such an unequal distribution of paralogs among groups of co-expressed genes extends to other processes during plant development is currently unknown.

## Conclusions

The results of our transcriptomics analysis of flower development, which covered most stages from the time of initiation until maturation, shows that the formation of flowers involves the differential expression of at least a quarter of the genes in the *Arabidopsis* genome. While many gene expression changes occur late in development and are likely due to the activation of specific gene sets in developing pollen and - to a lesser extent - ovules, genes with regulatory functions often exhibit intermittent expression during early and late floral stages. Through computational analyses, we have been able to assign functions to groups of co-expressed genes and to provide temporal information on when these processes likely occur during the almost two weeks during which flowers develop from a small number of meristematic cells into a highly complex structure with different organs, tissues and cell types. Using binding data for selected floral organ identity factors, we have further demonstrated that the results of our transcriptomics experiment can help to interpret and mine datasets from genome-wide localization studies. Our data also provide an important component of a gene expression map for flower development. Through the use of techniques such as Translating Ribosome Affinity Purification (TRAP) [11] or Isolation of Nuclei Tagged in specific Cell Types (INTACT) [55], it should be possible to extend this map by introducing detailed spatial information on gene expression for all floral stages.

## Methods

### Plant material, plant growth, treatment conditions and tissue collection

Plants of genotype AP1_pro_:AP1-GR *ap1-1 cal-1* [13] were grown on a soil:vermiculite:perlite (3:1:1) mixture at 20 °C under constant illumination with cool white fluorescent light. Flower development was induced in ~four week-old plants as described in [9], using a solution containing 10 μM dexamethasone (Sigma-Aldrich), 0.01 % (v/v) ethanol and 0.015 % (v/v) Silwet L-77 (De Sangosse). Floral buds were harvested at different time-points after dexamethasone treatment as described in Fig. 1. Three sets of biologically independent samples were collected for microarray analysis.

### Microarray experiments

Microarray experiments were performed using Agilent whole-genome *Arabidopsis* microarrays. For each microarray hybridization, amplified and dye-labeled RNA samples from a given time-point was co-hybridized with dye-labeled RNA from a common reference sample. This common reference was generated by pooling equal amounts of RNA from the individual time-points from 2 of the 3 sets of independent samples. RNA extractions, amplification and labelling of RNA preparations, microarray hybridizations, as well as washing and scanning of microarrays were done as previously described [12, 13].

### Processing of microarray data

Microarray data were analyzed using the software package *limma* (Linear Models for Microarray Data) [56] implemented in *R*. Background correction was done using the *subtract* method and within array normalization was performed with the *loess* method [57]. Between array normalization was done using the *Aquantile* method. Probes within each array were averaged on a gene-level and filtered to remove entries that had expression values below the median value of negative control probes. Linear models were fitted to the data using the *lmscFit* function. Correlograms were generated using the *R* package *corrgram*. Statistics for differential expression were first calculated using the *ebayes* function within *limma*. Genes with a *p*-value (after false discovery rate adjustment using the Benjamini-Hochberg procedure) below 0.01 were considered as differentially expressed. Because this analysis led to a very large number of differentially expressed genes that may not reflect true gene regulatory events (see Results and Discussion), we next compared gene expression between consecutive or near-by time-points using *ebayes*. To this end, we conducted all possible contrasts between time-points that lay within a 2-d interval (see Table S1 in Additional file 1). In order to be called as differentially expressed, genes were required to exhibit a *p*-value below 0.01 after adjustment for false discovery rate across the experiment and a fold-change in expression of 1.7 or greater.

*K*-means clustering was performed in *R* using scaled log_2_-transformed ratios of expression averaged across each replicate across all time-points for each gene, separating differentially expressed genes into 15 clusters on the basis of the similarity of the pattern of their temporal expression. The number of clusters was chosen heuristically based on the elbow method, which aims at maximizing the amount of variance explained while minimizing the number of clusters chosen. To this end, we compared, using the *kmeans* function implemented in *R*, the between-cluster sum-of-squares to the total sum-of-squares for different values for *k* (ranging from 2 to 200). We then plotted the data and selected a value for *k* in the ‘elbow’ of the plot.

### Comparison of expression data with data from an *Arabidopsis* gene expression atlas

Genes assigned to each *k*-means cluster were compared to a previously described [13] *Arabidopsis* gene expression atlas, which is based on published transcriptomics datasets for floral and non-floral tissues, to identify trends in tissue-specific expression within each cluster. This tissue atlas was also used to identify the tissues where genes within a cluster had their highest and lowest expression levels in order to investigate the correlation of changes in temporal expression within developing tissues.

### Gene ontology analysis

Gene Ontology analysis was performed using *PlantGSEA* [58]. Statistical significance calculations were performed with a Fisher’s exact test using False Discovery Rate adjustment method from Benjamini and Yekutieli [59] with a *p*-value cut off of 0.05.

### Identification of paralogs

All known protein sequences from *Arabidopsis* were individually aligned against the sequences from the entire proteome of *Arabidopsis* using *blastp* to select alignments with an E-value cut off of 1x10^-20^ and which covered 80 % of the query sequence [60]. The top 5 non-reciprocal alignments were retained as potential paralogs. Using this information, we determined the percentage of paralogs within each of the 15 clusters of differentially expressed genes described in Fig. 3. To test whether paralogs were significantly enriched in the clusters, we conducted the following background calculation: we first generated, for each cluster, two groups of genes drawn randomly either from the list of 7,405 differentially expressed genes or from genes present on the microarrays used in this study. Both groups contained 100 sets of genes each and the number of genes in a set was identical to the size of a cluster. We then calculated the mean percentage and standard deviations for paralogs in each of the groups and compared them to the percentage of paralogs we had identified in a corresponding cluster. Clusters with percentage values that were beyond three standard deviations from the random gene groups were considered significantly different.

### Comparison of expression data with data from genome-wide localization studies

Data from genome-wide localization studies were contrasted with each of the 15 *k*-means clusters to determine the frequency with which genes identified as being bound by the transcription factors AP1 and SEP3 [14], as well as by AP3, PI, and AG [12, 13], occurred in each cluster. This was contrasted against the frequencies with which bound genes occurred in randomized but equally-sized clusters of genes drawn from the 7,405 differentially expressed genes identified in the time-course experiment.

### Availability of supporting data

The data sets supporting the results of this article are included within the article (and its additional files). Microarray data have been deposited with the Gene Expression Omnibus (GEO) repository (at http://www.ncbi.nlm.nih.gov/) under GSE64581.

## Additional files

Additional file 1:
**Additional figures, tables and references.** Tables and figures in this file are referred to as Table S1-S3 and Figure S1-S4 in the main text. Additional file 2:
**Excel spreadsheet containing 7,405 genes identified as differentially expressed in this study.** Gene identifiers, aliases, descriptions, and their assignment to one of the fifteen *k*-means clusters are indicated. Also shown are the log_2_-transformed expression ratios and adjusted *p*-values for each contrast between time-points (‘T’). Additional file 3:
**Excel spreadsheet listing differentially expressed genes also identified in related studies.** Gene identifiers, aliases, descriptions, and their assignment to one of the fifteen *k*-means clusters are indicated. Datasets are described in Table S2 in Additional file 1. For the study by Wellmer et al. (2004) [16], the assignment of genes to one of the four types of floral organs is listed. For the study by Wellmer et al. (2006) [9], the assignment of genes to clusters (A-E) of co-expressed genes is shown. For the study by Gomez-Mena et al. [10], the presence or absence of genes among genes up- or down-regulated after AG-GR activation is indicated. For all other studies, *p*-values for genes from the related studies are shown. Additional file 4:
**Mapping groups of co-expressed genes onto an** ***Arabidopsis*** **gene expression atlas.** Expression data for an *Arabidopsis* gene expression atlas were obtained for genes assigned to each of the 15 *k*-means clusters and hierarchical clustering was performed. Individual tissue and organ samples of the gene expression atlas [12] are indicated. Additional file 5:
**Excel spreadsheet containing Gene Ontology terms identified as enriched in the dataset.** GO terms and adjusted *p*-values indicating a significant enrichment are shown for all *k*-means clusters. Additional file 6:
**Excel spreadsheet containing information on DEGs with binding sites for floral organ identity factors.** The gene identifiers, aliases, descriptions, and their assignment to one of the fifteen *k*-means clusters are indicated. Furthermore, the presence of binding sites for floral organ identity factors in the putative promoters of the genes are shown. To this end, data for AP1 and SEP3 from Pajoro et al. [14], for AP3 and PI from Wuest et al. [12], and for AG from O’Maoileidigh et al. [13] have been used. Whether or not a gene has been described previously [12, 13] as a direct target of AP3, PI, and AG is shown as well. Additional file 7:
**Microsoft Excel spreadsheet containing information relating to paralogs identified in groups of co-expressed genes.** For each gene identified as having paralogs in a given *k*-means cluster (as indicated), the gene identifiers, aliases, and descriptions, as well as the paralogous genes are shown.
