# Supplementary material for: Patterns of gene expression during Arabidopsis flower development from the time of initiation to maturation
Source: BMC Genomics. 2015 Jul 1;16(1):488. doi: 10.1186/s12864-015-1699-6 (PMC4488132; doi:10.1186/s12864-015-1699-6)
Supplement: Additional file 1: — Additional figures, tables and references. Tables and figures in this file are referred to as Table S1-S3 and Figure S1-S4 in the main text. [file 12864_2015_1699_MOESM1_ESM.pdf]

ADDITIONAL FILE 1

Content:

1. Supplemental Figures

2. Supplemental Tables

3. Supplemental References

1. SUPPLEMENTAL FIGURES

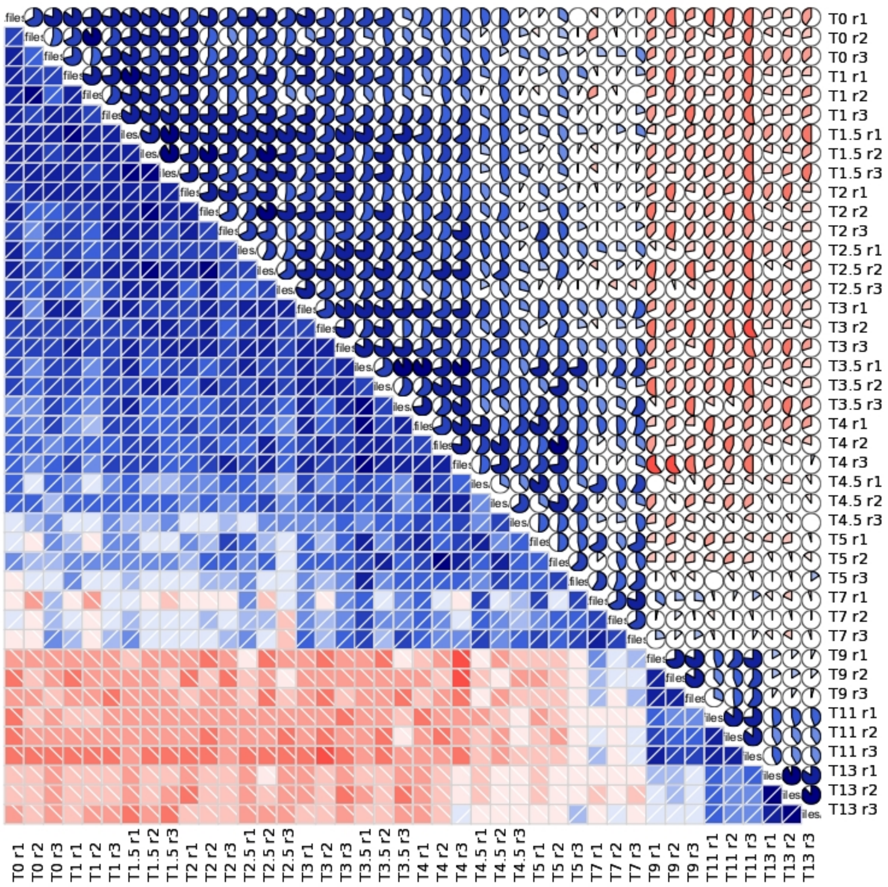

Figure S1. Reproducibility of microarray experiments.

The correlogram shown is based on the comparisons of *M* values ($\log_2$ (expression in sample/expression in common reference)) across all microarray datasets used for this study. Time-points ('T') and data from the three sets of samples ('r1', 'r2' and 'r3') are indicated. Fullness of pie charts in the upper panels represents the degree of correlation. Blue shades indicate a positive correlation and red shades indicate a negative correlation in both panels. The correlogram indicates a trend for replicate slides to correlate highly, and for later time points to differ significantly from early time points.

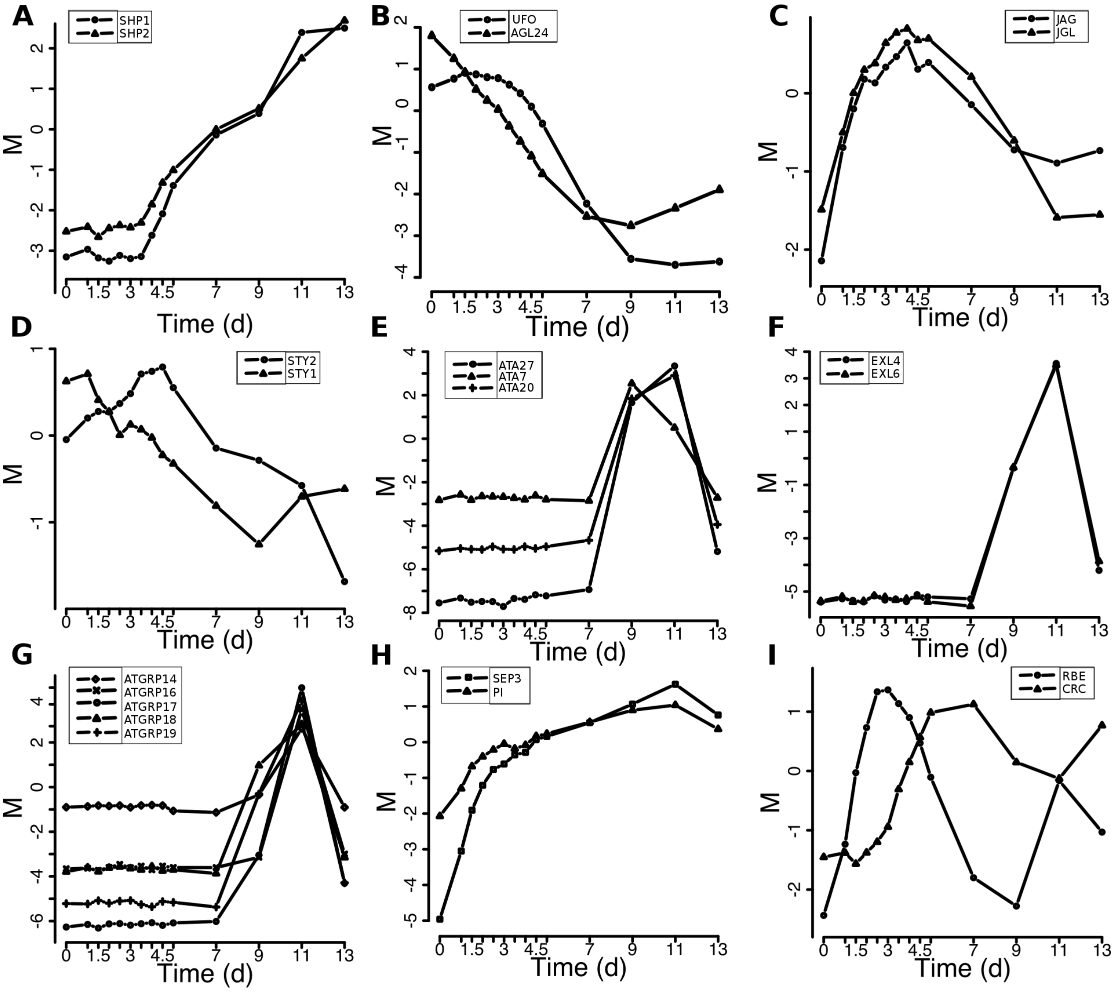

Figure S2. **Expression profiles of genes with known roles in flower development.**

(A-I) *M* values ($\log_2$ (expression in sample/expression in common reference)) are shown for selected floral regulators (as indicated) for all time-points. Lines represent average values for three replicate experiments.

(A) Up-regulation of *SHATTERPROOF1* (*SHP1*) and *SHP2* towards the end of early flower development is in agreement with the activation of these genes in carpel primordia around stage 6 [1, 2].

(B) Repression of *UNUSUAL FLORAL ORGANS* (*UFO*) from day 4 of the experiment is in agreement with its reported gradual down-regulation in flowers of early to intermediate stages [3]. Repression of *AGAMOUS-LIKE24* (*AGL24*) during

early flower development has been described and depends at least in part on AP1 activity [4, 5].

(C) Expression of *JAGGED* (*JAG*) and its paralog *JAGGED-LIKE/NUBBIN* (*JGL/NUB*) increases during early floral stages and gradually decreases at more mature stages, as previously described [6-8].

(D) Expression levels for *STYLISH1* (*STY1*) and (*STY2*) changed only moderately, in agreement with the prolonged expression of these genes in different regions of the developing flower from early stages onward [9].

(E) The genes *ARABIDOPSIS THALIANA ANTHER7* (*ATA7*), *ATA20* and *ATA27* are all expressed during microsporogenesis, which takes place around day 9 of the time-course experiment. The expression of *ATA20* and *ATA27* has been reported to be prolonged when compared to *ATA7* [10], in agreement with the expression profiles we have obtained.

(F-G) The extracellular lipases EXL4 and 6 and the oleosins GLYCINE RICH PROTEIN14 (GRP14), 16, 17, 18, and 19 are abundant components of the pollen coat proteome [11]. In agreement with this, we detected an expression peak for these genes at the 11 d time-point, when the late stages of pollen formation occur.

(H) In agreement with the expression profiles shown, the floral organ identity genes *SEP3* and *PI* are activated during early flower development and remain expressed in developing floral organs [12, 13].

(I) The rapid down-regulation observed for *RABBIT EARS* (*RBE*) is in agreement with its expression in petal primordia until stage 6 [14]. Up-regulation of *CRABS CLAW* (*CRC*) during early flower development and prolonged expression in carpel primordia and nectaries has been described previously [15].

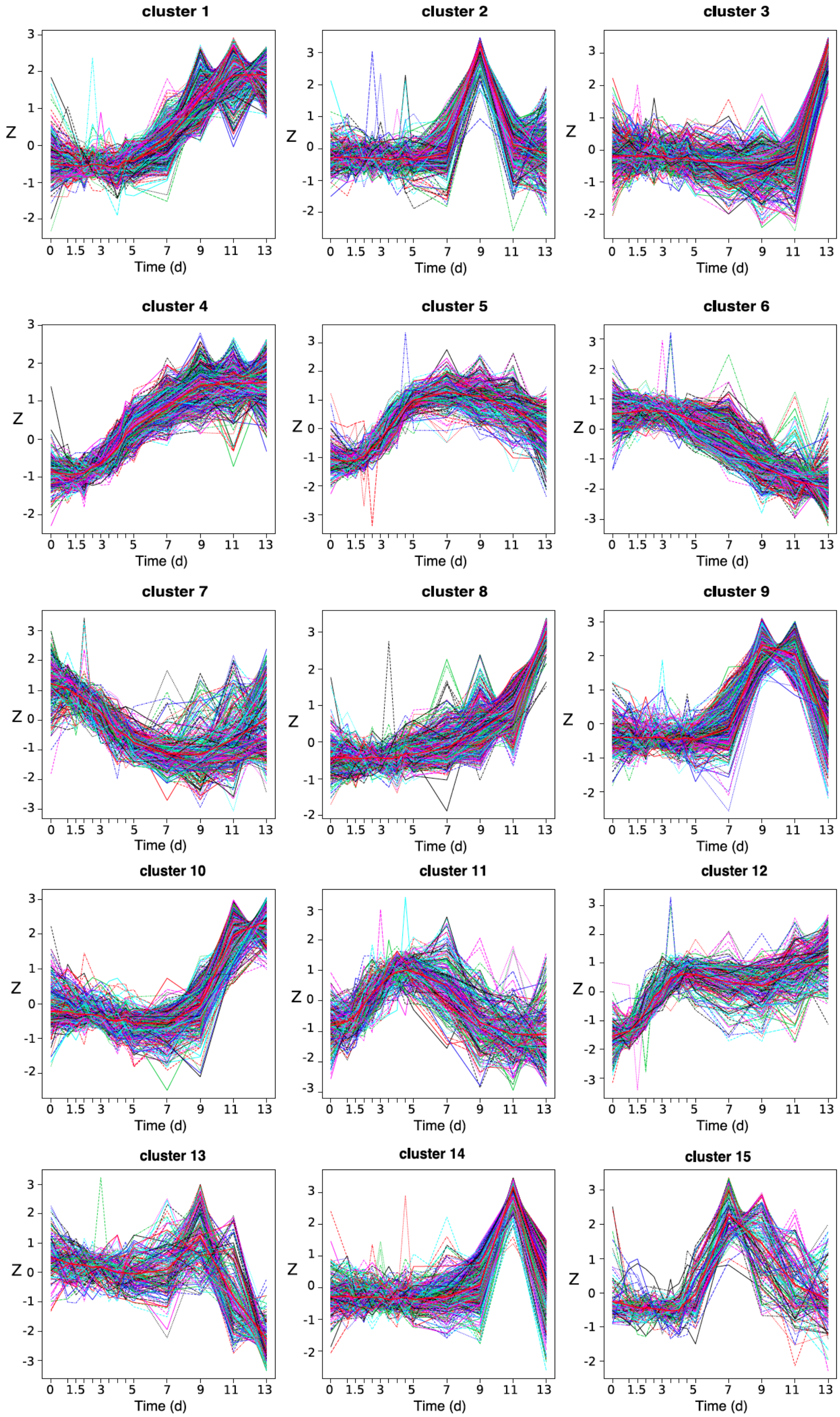

Figure S3. Identification of groups of co-expressed genes among 7,405 differentially expressed genes identified in the time-course experiment.

K-means clustering (*k*=15) was used to group genes based on their *z*-scores. Average *z*-scores are shown through a red line for each of the 15 clusters.

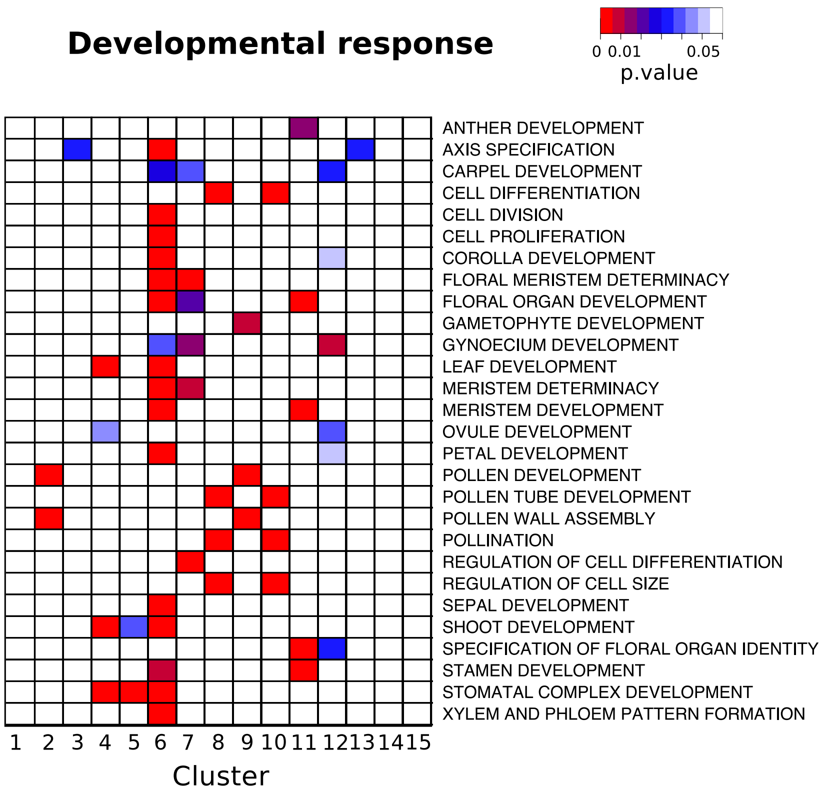
Developmental response
0 0.01 0.05
p.value
ANTHER DEVELOPMENT
AXIS SPECIFICATION
CARPEL DEVELOPMENT
CELL DIFFERENTIATION
CELL DIVISION
CELL PROLIFERATION
COROLLA DEVELOPMENT
FLORAL MERISTEM DETERMINACY
FLORAL ORGAN DEVELOPMENT
GAMETOPHYTE DEVELOPMENT
GYNOECIUM DEVELOPMENT
LEAF DEVELOPMENT
MERISTEM DETERMINACY
MERISTEM DEVELOPMENT
OVULE DEVELOPMENT
PETAL DEVELOPMENT
POLLEN DEVELOPMENT
POLLEN TUBE DEVELOPMENT
POLLEN WALL ASSEMBLY
POLLINATION
REGULATION OF CELL DIFFERENTIATION
REGULATION OF CELL SIZE
SEPAL DEVELOPMENT
SHOOT DEVELOPMENT
SPECIFICATION OF FLORAL ORGAN IDENTITY
STAMEN DEVELOPMENT
STOMATAL COMPLEX DEVELOPMENT
XYLEM AND PHLOEM PATTERN FORMATION
1 2 3 4 5 6 7 8 9 10 11 12 13 14 15
Cluster

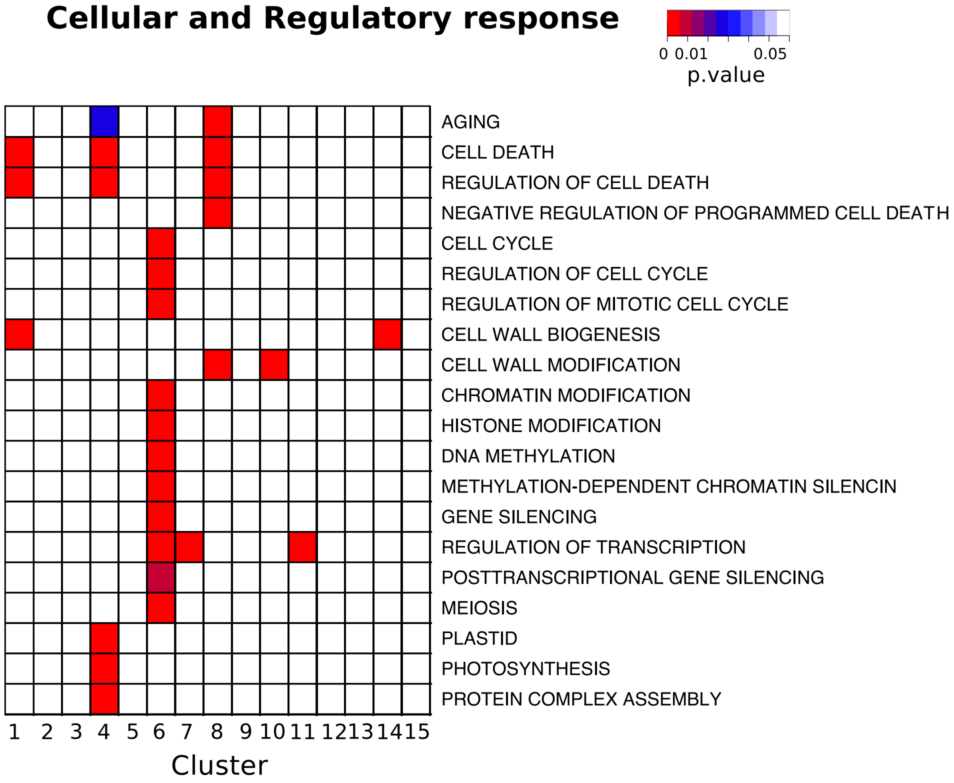
Cellular and Regulatory response
0 0.01 0.05
p.value
AGING
CELL DEATH
REGULATION OF CELL DEATH
NEGATIVE REGULATION OF PROGRAMMED CELL DEATH
CELL CYCLE
REGULATION OF CELL CYCLE
REGULATION OF MITOTIC CELL CYCLE
CELL WALL BIOGENESIS
CELL WALL MODIFICATION
CHROMATIN MODIFICATION
HISTONE MODIFICATION
DNA METHYLATION
METHYLATION-DEPENDENT CHROMATIN SILENCIN
GENE SILENCING
REGULATION OF TRANSCRIPTION
POSTTRANSCRIPTIONAL GENE SILENCING
MEIOSIS
PLASTID
PHOTOSYNTHESIS
PROTEIN COMPLEX ASSEMBLY
1 2 3 4 5 6 7 8 9 10 11 12 13 14 15
Cluster

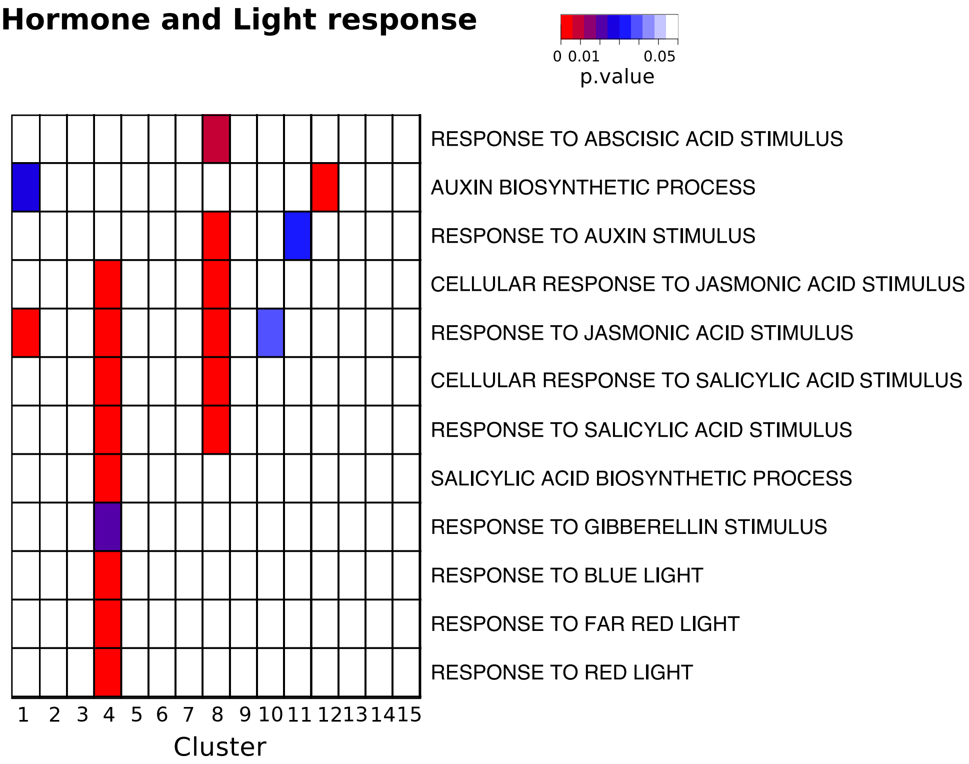

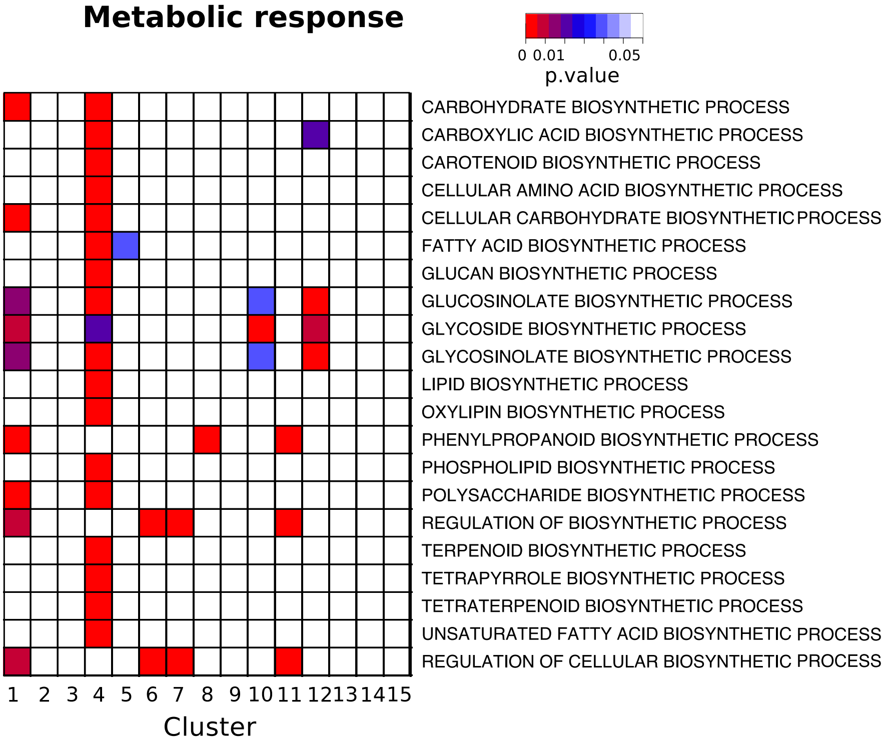

Figure S4. Gene Ontology terms enriched in the dataset.

Adjusted *p*-values for selected GO terms related to (from top to bottom) developmental responses; cellular and regulatory responses; hormone and light responses; and metabolic responses are indicated for each cluster through color-

coding (see bars at the top right of each heatmap for colors used). For a full list of GO terms enriched in the dataset, see Additional File 5. Several of the GO terms shown are also part of Figure 5. They were included here again to allow a direct comparison with other related terms.

2. SUPPLEMENTAL TABLES

Table S1: Differentially expressed genes identified in the different contrasts. The number of differentially expressed genes ('No. of DEG') identified in the different contrasts between time-points ('T') is indicated. In the lower section of the table, the results for the contrasts between time-points with 2-d intervals are shown. In this case, the number of up- and down-regulated genes is listed as well.

Contrast	No. of DEG	Contrast	No. of DEG
**T1.5 vs. T1**	25	**T1 vs. T0**	115
**T2 vs. T1.5**	22	**T2 vs. T1**	134
**T2.5 vs. T2**	36	**T2.5 vs. T1.5**	186
**T3 vs. T2.5**	24	**T3 vs. T2**	183
**T3.5 vs. T3**	59	**T3.5 vs. T2.5**	245
**T4 vs. T3.5**	28	**T4 vs. T3**	268
**T4.5 vs. T4**	94	**T4.5 vs. T3.5**	316
**T5 vs. T4.5**	13	**T5 vs. T4**	323
**Contrast**	**No. of DEG**	**up-regulated**	**down-regulated**
**T2 vs. T0**	420	231	189
**T3 vs. T1**	579	430	149
**T3.5 vs. T1.5**	800	593	207
**T4 vs. T2**	854	623	231
**T4.5 vs.T2.5**	924	725	199
**T5 vs. T3**	1050	796	254
**T7 vs. T5**	868	705	163
**T9 vs. T7**	3125	2433	692
**T11 vs. T9**	3198	1987	1211
**T13 vs. T11**	4099	2309	1790

Table S2: **Comparison of differentially expressed genes with published datasets.** The 7,405 genes we identified as differentially expressed during flower development were compared against the datasets from several related studies. The number of genes in the different datasets are shown in column 'Number of genes in dataset'. The intersections observed and expected by chance between the 7,405 DEGs and the individual datasets are given in columns 'Intersection – observed' and 'Intersection – expected', respectively. Datasets from the following studies were considered:

- Jiao and Meyerowitz ('Jiao') used TRAP-Seq to determine gene expression in the expression domains of *AP1*, *AP3* and *AG*, respectively, during early flower development [16].
- Mantegazza et al. ('Mantegazza') used laser capture microdissection and RNA-Seq to study gene expression in very young floral buds [17]. The separate datasets stem from the contrasts of the different tissues sampled: FM - floral meristem; IM - inflorescence meristem; ST3 - stage 3.
- Pajoro et al. ('Pajoro') used the same floral induction system as in the present study to monitor gene expression at selected early to intermediate stages (2, 4, and 8 d after dexamethasone treatment) using microarray analysis [18]. The separate datasets stem from the contrasts of the different time-points sampled. IM - inflorescence meristem.
- Wellmer et al. ('Wellmer 2004') compared gene expression in whole inflorescences of floral homeotic mutants and the wild type [19].
- Wellmer et al. ('Wellmer 2006') used a floral induction system to study gene expression during early flower development [20].
- Gomez-Mena et al. ('Gomez-Mena') used an AG-GR fusion protein in an *ap1 cal* double-mutant background to identify genes that are up- or down-regulated in early-stage flowers containing reproductive floral organs only [21].

Dataset	Number of genes in dataset	Intersection - observed	Intersection - expected
Jiao (AG)	715	370	184
Jiao (AP1)	214	75	55
Jiao (AP3)	1744	536	449
Mantegazza (FM/IM)	46	31	12
Mantegazza (FM/ST3)	171	124	44
Mantegazza (ST3/IM)	178	123	46
Pajoro (IM/2d)	4024	1599	1035
Pajoro (2d/4d)	2692	1096	692
Pajoro (4d/8d)	4776	2150	1229
Wellmer 2004	1453	1240	374
Wellmer 2006	1653	987	425
Gomez-Mena (Up)	150	106	39
Gomez-Mena (Down)	66	45	17

Table S3: Occurrence of paralogs in groups of co-expressed genes.

The percentage of paralogs identified in each of the 15 *k*-means clusters is shown in column '% Cluster'. For each cluster, the mean percentage values of paralogs identified in 100 equally sized groups of genes taken randomly from the 7,405 DEGs or from the *Arabidopsis* genome are listed in columns '% DEG' and '% Genome', respectively. Standard deviations of these background calculations are given in columns 'SD DEG' and 'SD Genome', respectively. Ratios between the percentage of paralogs identified in each cluster and the mean percentage values of paralogs identified in 100 equally-sized groups of genes taken randomly from the 7,405 DEGs or from the *Arabidopsis* genome are listed in columns 'Ratio Cluster/DEG' and 'Ratio Cluster/Genome', respectively.

Cluster	% Cluster	% DEG	SD DEG	% Genome	SD Genome	Ratio Cluster /DEG	Ratio Cluster/ Genome
1	26.1	14.7	1.5	6.5	1.1	1.8	4.0
2	32.4	11.9	1.6	5.3	1.2	2.7	6.1
3	31.6	16.8	1.4	7.6	1.0	1.9	4.1
4	23.6	12.3	1.5	5.7	1.1	1.9	4.1
5	21.0	5.5	1.9	2.3	1.2	3.8	9.0
6	31.0	12.0	1.5	5.3	1.2	2.6	5.9
7	16.5	5.6	1.6	2.6	1.2	2.9	6.3
8	23.4	13.1	1.6	6.0	1.2	1.8	3.9
9	24.5	12.2	1.7	5.6	1.0	2.0	4.4
10	21.8	10.6	1.7	4.7	1.1	2.1	4.6
11	18.8	4.8	1.7	2.0	1.2	3.9	9.4
12	15.6	4.3	2.0	1.7	1.2	3.6	9.4
13	7.3	3.6	1.7	1.4	1.2	2.0	5.1
14	17.8	11.2	1.6	5.0	1.3	1.6	3.5
15	11.7	2.8	1.9	1.2	1.3	4.2	9.4

3. SUPPLEMENTAL REFERENCES

1. Flanagan CA, Hu Y, Ma H: **Specific expression of the AGL1 MADS-box gene suggests regulatory functions in Arabidopsis gynoecium and ovule development**. *Plant J* 1996, **10**(2):343-353.
2. Savidge B, Rounsley SD, Yanofsky MF: **Temporal relationship between the transcription of two Arabidopsis MADS box genes and the floral organ identity genes**. *Plant Cell* 1995, **7**(6):721-733.
3. Lee I, Wolfe DS, Nilsson O, Weigel D: **A LEAFY co-regulator encoded by UNUSUAL FLORAL ORGANS**. *Curr Biol* 1997, **7**(2):95-104.
4. Liu C, Zhou J, Bracha-Drori K, Yalovsky S, Ito T, Yu H: **Specification of Arabidopsis floral meristem identity by repression of flowering time genes**. *Development* 2007, **134**(10):1901-1910.
5. Kaufmann K, Wellmer F, Muino JM, Ferrier T, Wuest SE, Kumar V, Serrano-Mislata A, Madueno F, Krajewski P, Meyerowitz EM *et al*: **Orchestration of floral initiation by APETALA1**. *Science* 2010, **328**(5974):85-89.
6. Dinneny JR, Weigel D, Yanofsky MF: **NUBBIN and JAGGED define stamen and carpel shape in Arabidopsis**. *Development* 2006, **133**:1645-1655.
7. Dinneny JR, Yadegari R, Fischer RL, Yanofsky MF, Weigel D: **The role of JAGGED in shaping lateral organs**. *Development* 2004, **131**(5):1101-1110.
8. Ohno CK, Reddy GV, Heisler MG, Meyerowitz EM: **The Arabidopsis JAGGED gene encodes a zinc finger protein that promotes leaf tissue development**. *Development* 2004, **131**(5):1111-1122.
9. Kuusk S, Sohlberg JJ, Long JA, Fridborg I, Sundberg E: **STY1 and STY2 promote the formation of apical tissues during Arabidopsis gynoecium development**. *Development* 2002, **129**(20):4707-4717.
10. Rubinelli P, Hu Y, Ma H: **Identification, sequence analysis and expression studies of novel anther-specific genes of Arabidopsis thaliana**. *Plant Mol Biol* 1998, **37**(4):607-619.
11. Mayfield JA, Fiebig A, Johnstone SE, Preuss D: **Gene families from the Arabidopsis thaliana pollen coat proteome**. *Science* 2001, **292**(5526):2482-2485.
12. Goto K, Meyerowitz EM: **Function and regulation of the Arabidopsis floral homeotic gene PISTILLATA**. *Genes Dev* 1994, **8**(13):1548-1560.
13. Mandel MA, Yanofsky M: **The *Arabidopsis AGL9* MADS-box gene is expressed in young flower promordia**. *Sex Plant Reprod* 1998, **11**:22-28.
14. Takeda S, Matsumoto N, Okada K: **RABBIT EARS, encoding a SUPERMAN-like zinc finger protein, regulates petal development in Arabidopsis thaliana**. *Development* 2004, **131**(2):425-434.
15. Bowman JL, Smyth DR: **CRABS CLAW, a gene that regulates carpel and nectary development in Arabidopsis, encodes a novel protein with zinc**

finger and helix-loop-helix domains. *Development* 1999, **126**(11):2387-2396.

16. Jiao Y, Meyerowitz EM: **Cell-type specific analysis of translating RNAs in developing flowers reveals new levels of control**. *Molecular systems biology* 2010, **6**:419.

17. Mantegazza O, Gregis V, Chiara M, Selva C, Leo G, Horner DS, Kater MM: **Gene coexpression patterns during early development of the native Arabidopsis reproductive meristem: novel candidate developmental regulators and patterns of functional redundancy**. *Plant J* 2014, **79**(5):861-877.

18. Pajoro A, Madrigal P, Muino JM, Matus JT, Jin J, Mecchia MA, Debernardi JM, Palatnik JF, Balazadeh S, Arif M *et al*: **Dynamics of chromatin accessibility and gene regulation by MADS-domain transcription factors in flower development**. *Genome Biol* 2014, **15**(3):R41.

19. Wellmer F, Riechmann JL, Alves-Ferreira M, Meyerowitz EM: **Genome-wide analysis of spatial gene expression in Arabidopsis flowers**. *Plant Cell* 2004, **16**(5):1314-1326.

20. Wellmer F, Alves-Ferreira M, Dubois A, Riechmann JL, Meyerowitz EM: **Genome-wide analysis of gene expression during early Arabidopsis flower development**. *PLoS Genet* 2006, **2**(7):e117.

21. Gomez-Mena C, de Folter S, Costa MM, Angenent GC, Sablowski R: **Transcriptional program controlled by the floral homeotic gene AGAMOUS during early organogenesis**. *Development* 2005, **132**(3):429-438.
