# Supplementary material for: Patterns of gene expression during Arabidopsis flower development from the time of initiation to maturation
Source: BMC Genomics. 2015 Jul 1;16(1):488. doi: 10.1186/s12864-015-1699-6 (PMC4488132; doi:10.1186/s12864-015-1699-6)
Supplement: Additional file 4: — Mapping groups of co-expressed genes onto an Arabidopsis gene expression atlas. Expression data for an Arabidopsis gene expression atlas were obtained for genes assigned to each of the 15 k-means clusters and hierarchical clustering was performed. Individual tissue and organ samples of the gene expression atlas [12] are indicated. [file 12864_2015_1699_MOESM4_ESM.pdf]

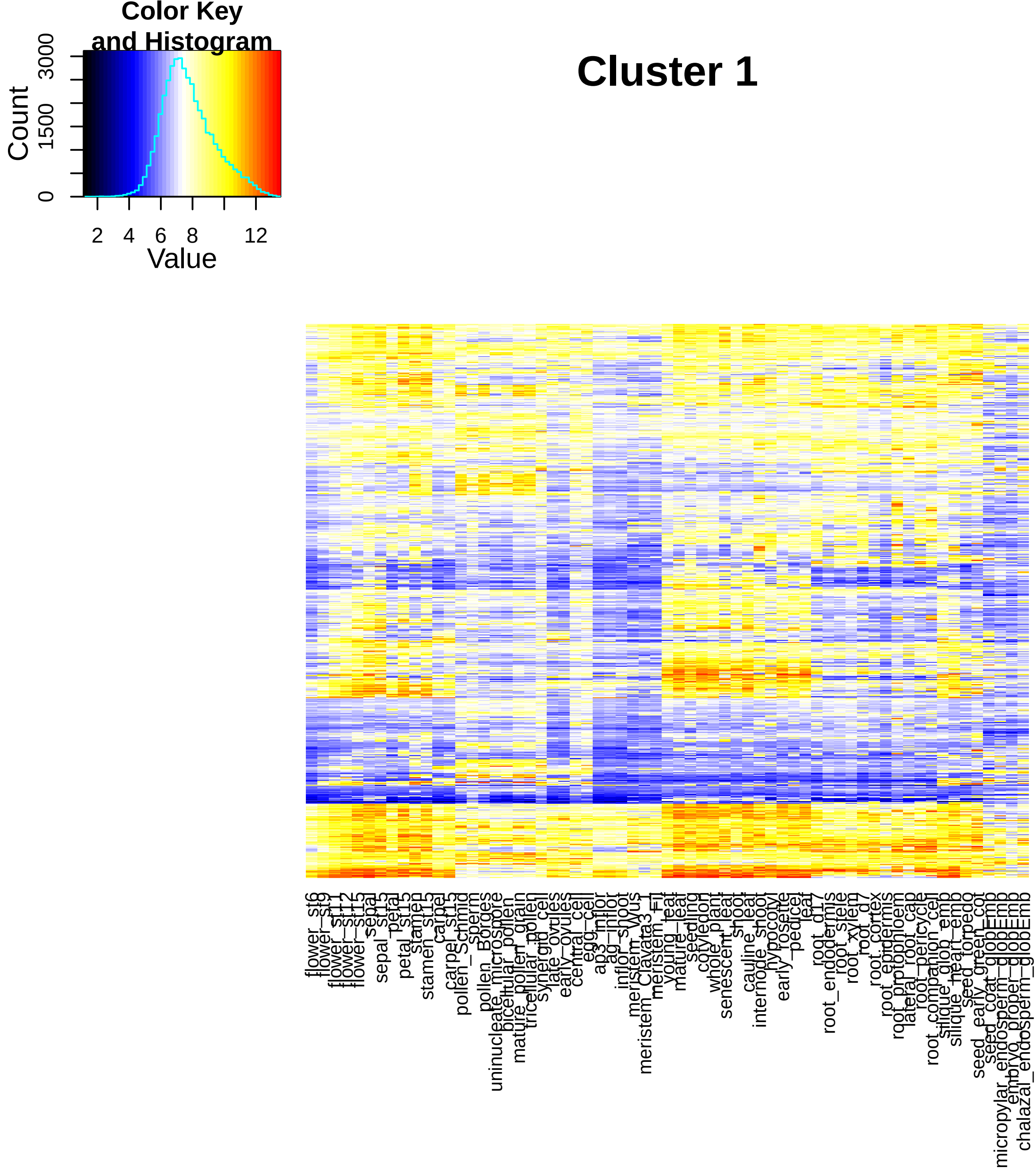
Color Key
and Histogram
Count
0
1500
3000
2
4
6
8
12
Value
Cluster 1
flower_st6
flower_st9
flower_st11
flower_st12
flower_st15
sepal
sepal_st15
petal
petal_st15
stamen
stamen_st15
carpel
carpel_st15
pollen_Schmid
sperm
pollen_Borges
uninucleate_microspore
bicellular_pollen
mature_pollen_grain
tricellular_pollen
synergid_cell
late_ovules
early_ovules
central_cell
egg_cell
ap3_inflor
ag_inflor
inflor_shoot
meristem_Wus
meristem_Clavata3
meristem_Fil
young_leaf
mature_leaf
seedling
cotyledon
whole_plant
senescent_leaf
shoot
cauline_leaf
internode_shoot
hypocotyl
early_rosette
pedicel
leaf
root_d17
root_endodermis
root_stele
root_xylem
root_d7
root_cortex
root_epidermis
root_protophloem
lateral_root_cap
root_pericycle
root_companion_cell
silique_glob_emb
silique_heart_emb
seed_torpedo
seed_early_green_cot
seed_coat_globEmb
micropylar_endosperm_globEmb
embryo_proper_globEmb
chalazal_endosperm_globEmb

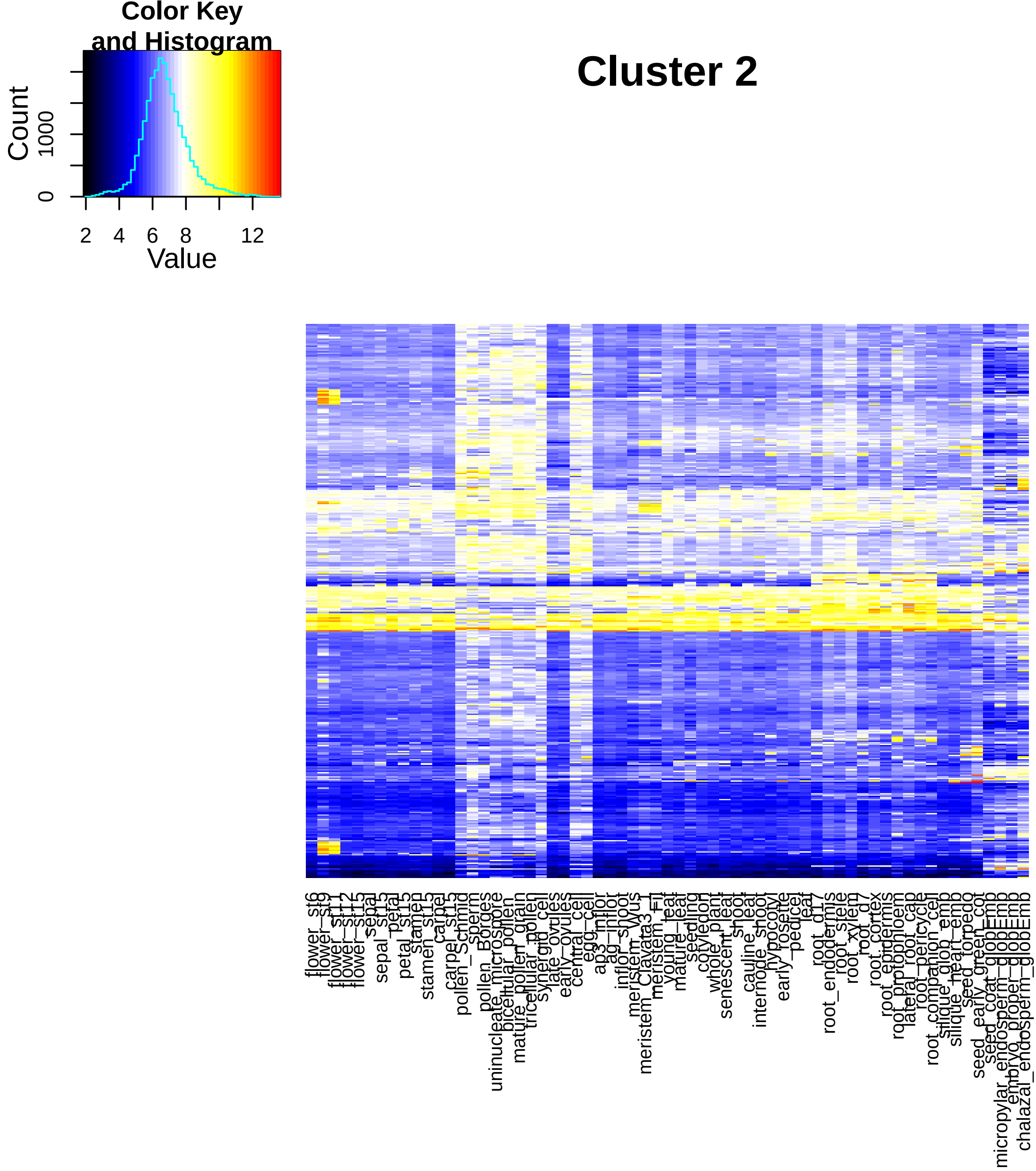
Color Key
and Histogram
Count
1000
0
2
4
6
8
12
Value
Cluster 2
flower_st6
flower_st9
flower_st11
flower_st12
flower_st15
sepal
sepal_st15
petal
petal_st15
stamen
stamen_st15
carpel
carpel_st15
pollen_Schmid
sperm
pollen_Borges
uninucleate_microspore
bicellular_pollen
mature_pollen_grain
tricellular_pollen
synergid_cell
late_ovules
early_ovules
central_cell
egg_cell
ap3_inflor
ag_inflor
inflor_shoot
meristem_Wus
meristem_Clavata3
meristem_Fil
young_leaf
mature_leaf
seedling
cotyledon
whole_plant
senescent_leaf
shoot
cauline_leaf
internode_shoot
hypocotyl
early_rosette
pedicel
leaf
root_d17
root_endodermis
root_stele
root_xylem
root_d7
root_cortex
root_epidermis
root_protophloem
lateral_root_cap
root_pericycle
root_companion_cell
silique_glob_emb
silique_heart_emb
seed_torpedo
seed_early_green_cot
seed_coat_globEmb
micropylar_endosperm_globEmb
embryo_proper_globEmb
chalazal_endosperm_globEmb

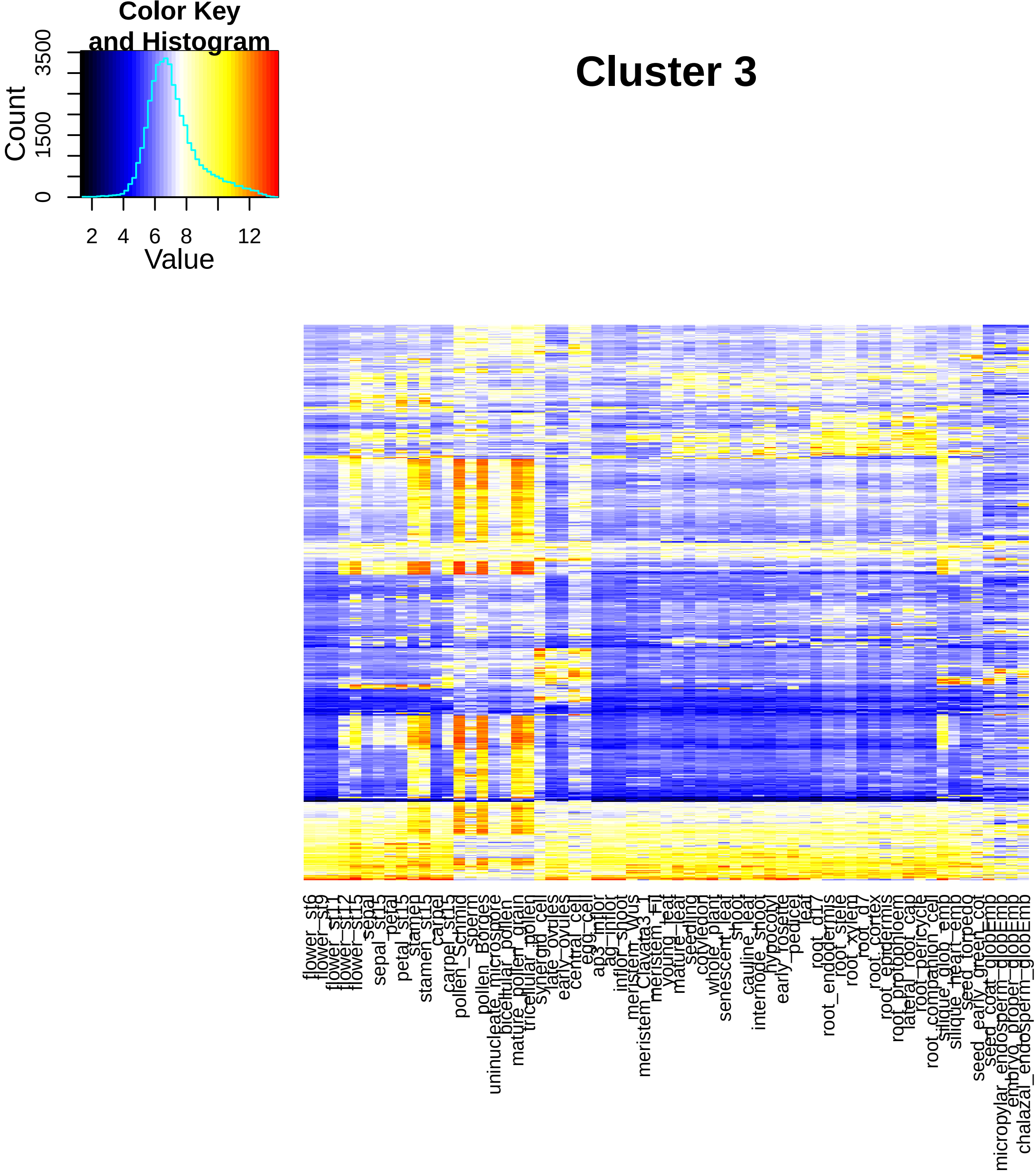
Color Key
and Histogram
Count
0
1500
3500
2
4
6
8
12
Value
Cluster 3
flower_st6
flower_st9
flower_st11
flower_st12
flower_st15
sepal
sepal_st15
petal
petal_st15
stamen
stamen_st15
carpel
carpel_st15
pollen_Schmid
sperm
pollen_Borges
uninucleate_microspore
bicellular_pollen
mature_pollen_grain
tricellular_pollen
synergid_cell
late_ovules
early_ovules
central_cell
egg_cell
ap3_inflor
ag_inflor
inflor_shoot
meristem_Wus
meristem_Clavata3
meristem_Fil
young_leaf
mature_leaf
seedling
cotyledon
whole_plant
senescent_leaf
shoot
cauline_leaf
internode_shoot
hypocotyl
early_rosette
pedicel
leaf
root_d17
root_endodermis
root_stele
root_xylem
root_d7
root_cortex
root_epidermis
root_protophloem
lateral_root_cap
root_pericycle
root_companion_cell
silique_glob_emb
silique_heart_emb
seed_torpedo
seed_early_green_cot
seed_coat_globEmb
micropylar_endosperm_globEmb
embryo_proper_globEmb
chalazal_endosperm_globEmb

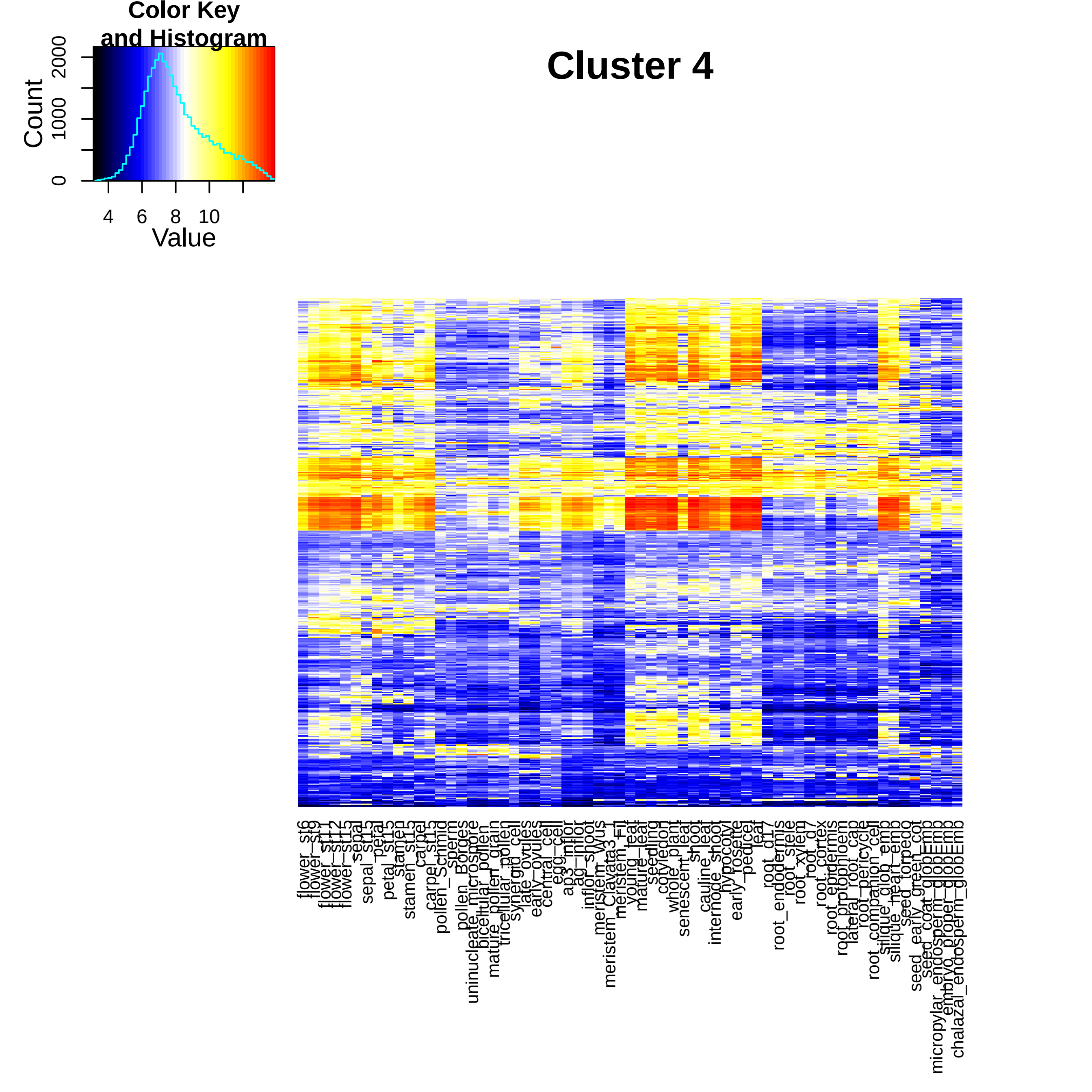
Color Key
and Histogram
Count
0
1000
2000
4
6
8
10
Value
Cluster 4
flower_st6
flower_st9
flower_st11
flower_st12
flower_st15
sepal
sepal_st15
petal
petal_st15
stamen
stamen_st15
carpel
carpel_st15
pollen_Schmid
sperm
pollen_Borges
uninucleate_microspore
bicellular_pollen
mature_pollen_grain
tricellular_pollen
synergid_cell
late_ovules
early_ovules
central_cell
egg_cell
ap3_inflor
ag_inflor
inflor_shoot
meristem_Wus
meristem_Clavata3
meristem_Fil
young_leaf
mature_leaf
seedling
cotyledon
whole_plant
senescent_leaf
shoot
cauline_leaf
internode_shoot
hypocotyl
early_rosette
pedicel
leaf
root_d17
root_endodermis
root_stele
root_xylem
root_d7
root_cortex
root_epidermis
root_protophloem
lateral_root_cap
root_pericycle
root_companion_cell
silique_glob_emb
silique_heart_emb
seed_torpedo
seed_early_green_cot
seed_coat_globEmb
micropylar_endosperm_globEmb
embryo_proper_globEmb
chalazal_endosperm_globEmb

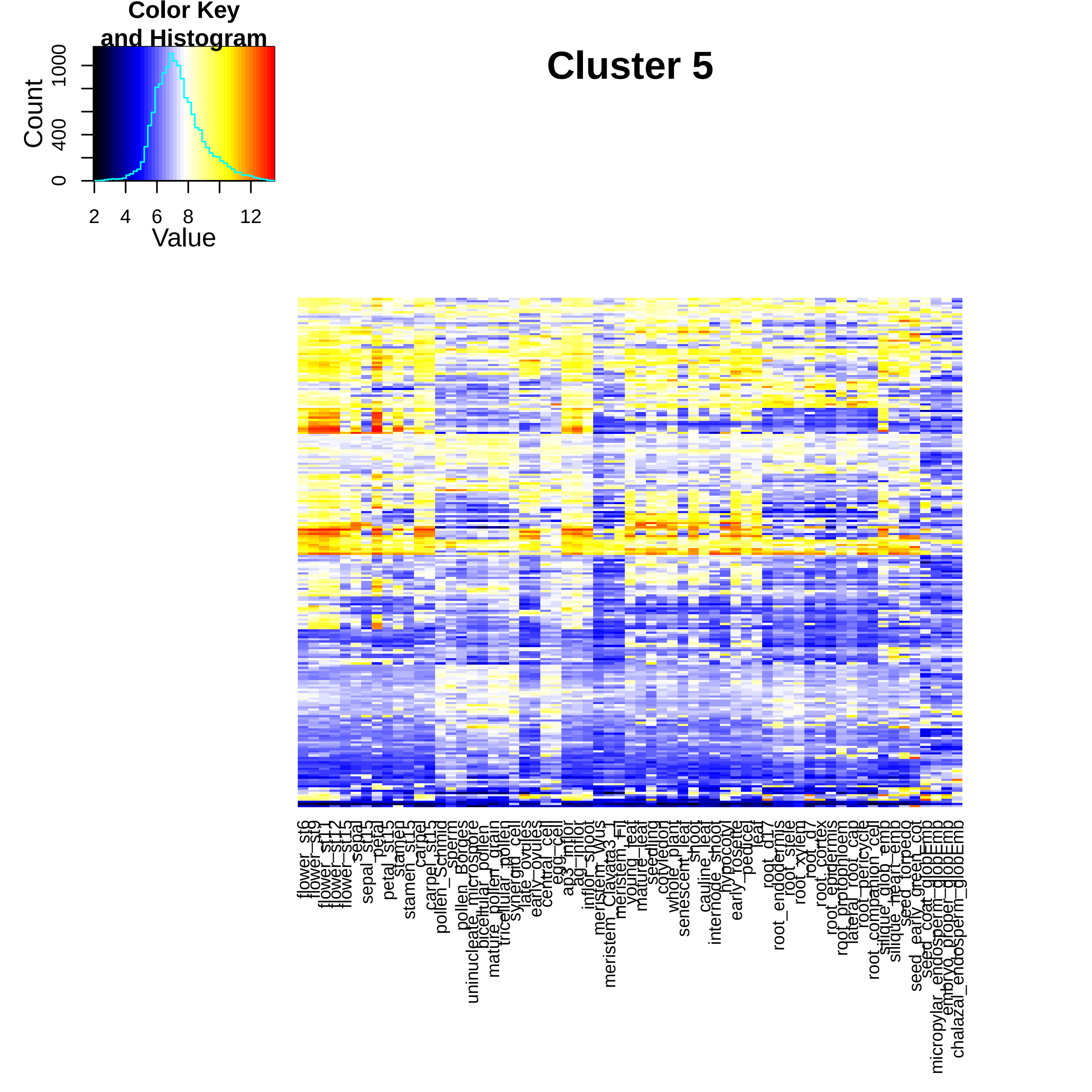

Color Key
and Histogram
Count
Value
0
400
1000
2
4
6
8
12
Cluster 5
flower_st6
flower_st9
flower_st11
flower_st12
flower_st15
sepal
sepal_st15
petal
petal_st15
stamen
stamen_st15
carpel
carpel_st15
pollen_Schmid
sperm
pollen_Borges
uninucleate_microspore
bicellular_pollen
mature_pollen_grain
tricellular_pollen
synergid_cell
late_ovules
early_ovules
central_cell
egg_cell
ap3_inflor
ag_inflor
inflor_shoot
meristem_Wus
meristem_Clavata3
meristem_Fil
young_leaf
mature_leaf
seedling
cotyledon
whole_plant
senescent_leaf
shoot
cauline_leaf
internode_shoot
hypocotyl
early_rosette
pedicel
leaf
root_d17
root_endodermis
root_stele
root_xylem
root_d7
root_cortex
root_epidermis
root_protophloem
lateral_root_cap
root_pericycle
root_companion_cell
silique_glob_emb
silique_heart_emb
seed_torpedo
seed_early_green_cot
seed_coat_globEmb
micropylar_endosperm_globEmb
embryo_proper_globEmb
chalazal_endosperm_globEmb

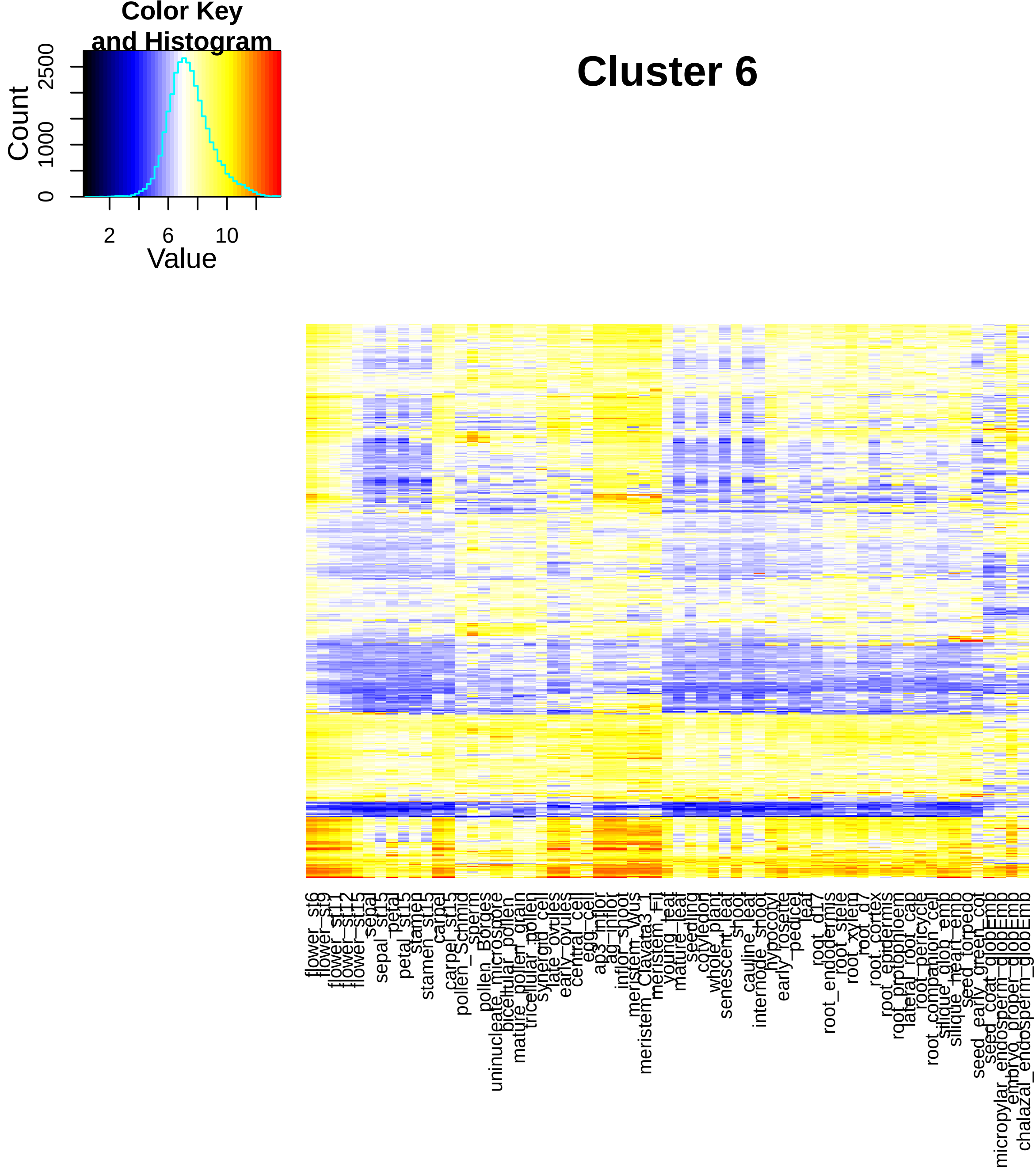
Color Key and Histogram
Count
0
1000
2500
2
6
10
Value
Cluster 6
flower_st6
flower_st9
flower_st11
flower_st12
flower_st15
sepal
sepal_st15
petal
petal_st15
stamen
stamen_st15
carpel
carpel_st15
pollen_Schmid
sperm
pollen_Borges
uninucleate_microspore
bicellular_pollen
mature_pollen_grain
tricellular_pollen
synergid_cell
late_ovules
early_ovules
central_cell
egg_cell
ap3_inflor
ag_inflor
inflor_shoot
meristem_Wus
meristem_Clavata3
meristem_Fil
young_leaf
mature_leaf
seedling
cotyledon
whole_plant
senescent_leaf
shoot
cauline_leaf
internode_shoot
hypocoty
early_rosette
pedicel
leaf
root_d17
root_endodermis
root_stele
root_xylem
root_d7
root_cortex
root_epidermis
root_protophloem
lateral_root_cap
root_pericycle
root_companion_cell
silique_glob_emb
silique_heart_emb
seed_torpedo
seed_early_green_cot
seed_coat_globEmb
micropylar_endosperm_globEmb
embryo_proper_globEmb
chalazal_endosperm_globEmb

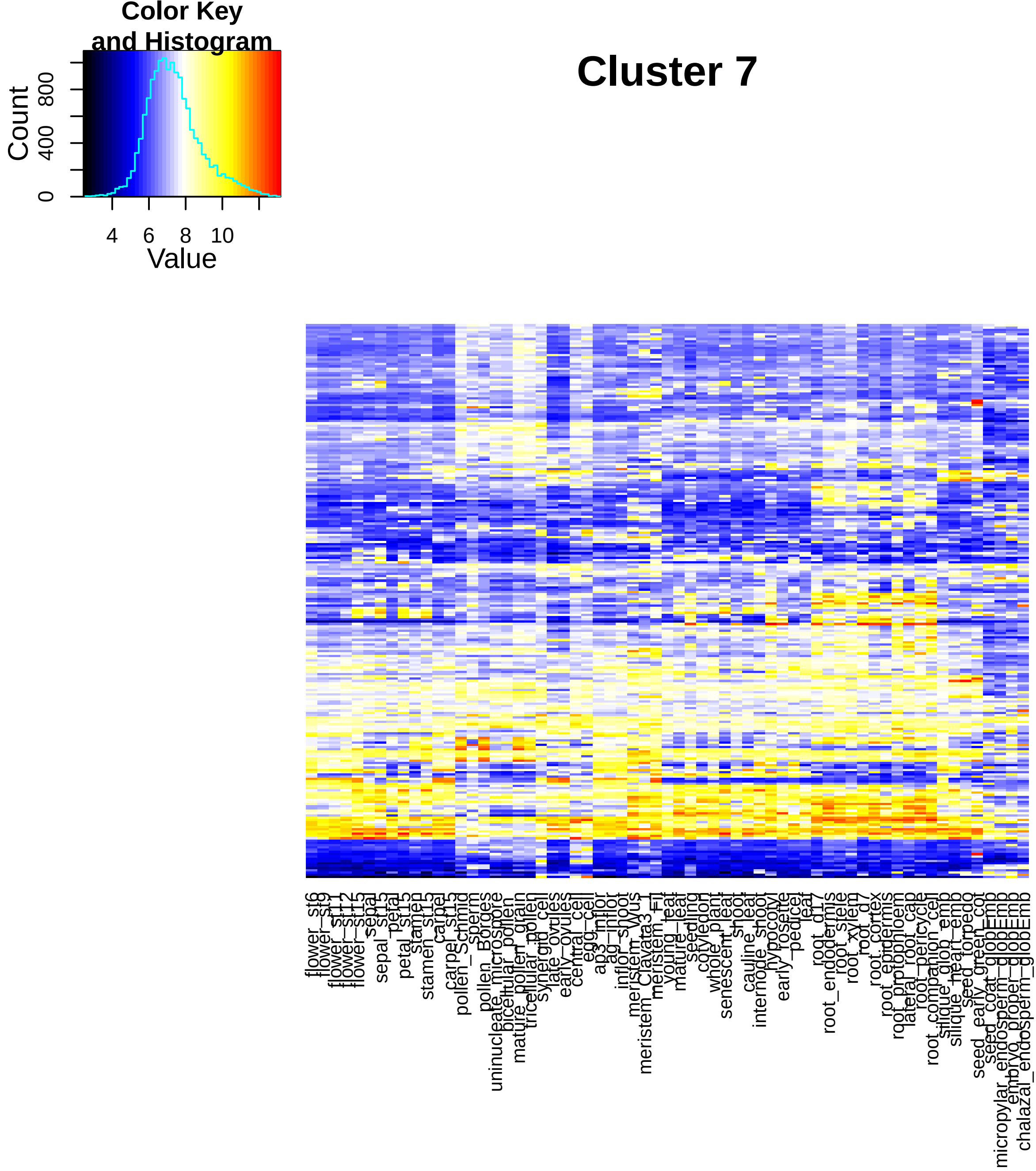

Color Key
and Histogram
Count
0
400
800
4
6
8
10
Value
Cluster 7
flower_st6
flower_st9
flower_st11
flower_st12
flower_st15
sepal
sepal_st15
petal
petal_st15
stamen
stamen_st15
carpel
carpel_st15
pollen_Schmid
sperm
pollen_Borges
uninucleate_microspore
bicellular_pollen
mature_pollen_grain
tricellular_pollen
synergid_cell
late_ovules
early_ovules
central_cell
egg_cell
ap3_inflor
ag_inflor
inflor_shoot
meristem_Wus
meristem_Clavata3
meristem_Fil
young_leaf
mature_leaf
seedling
cotyledon
whole_plant
senescent_leaf
shoot
cauline_leaf
internode_shoot
hypocoty
early_rosette
pedicel
leaf
root_d17
root_endodermis
root_stele
root_xylem
root_d7
root_cortex
root_epidermis
root_protophloem
lateral_root_cap
root_pericycle
root_companion_cell
silique_glob_emb
silique_heart_emb
seed_torpedo
seed_early_green_cot
seed_coat_globEmb
micropylar_endosperm_globEmb
embryo_proper_globEmb
chalazal_endosperm_globEmb

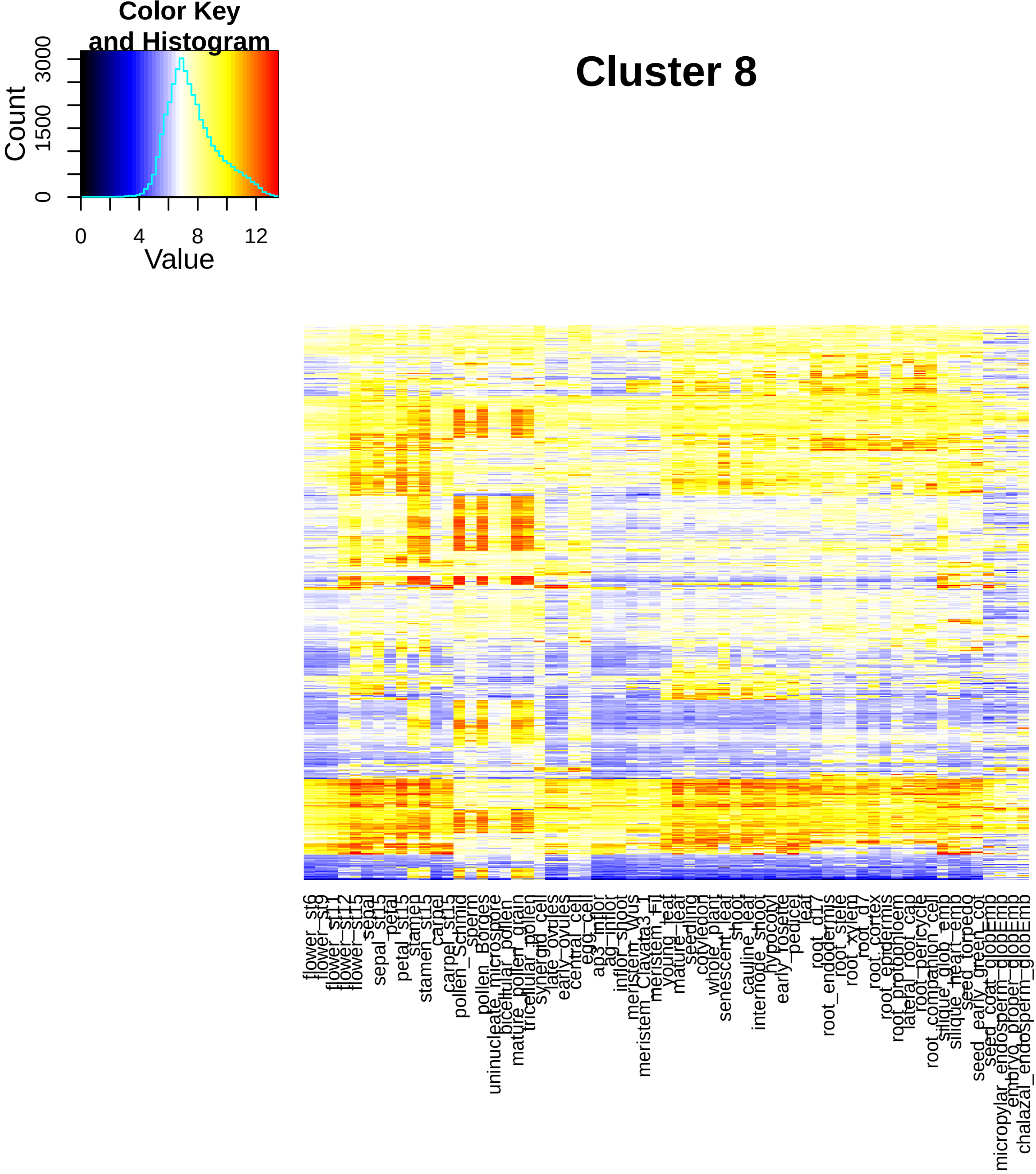
Color Key and Histogram
Count
0
1500
3000
0
4
8
12
Value
Cluster 8
flower_st6
flower_st9
flower_st11
flower_st12
flower_st15
sepal
sepal_st15
petal
petal_st15
stamen
stamen_st15
carpel
carpel_st15
pollen_Schmid
sperm
pollen_Borges
uninucleate_microspore
bicellular_pollen
mature_pollen_grain
tricellular_pollen
synergid_cell
late_ovules
early_ovules
central_cell
egg_cell
ap3_inflor
ag_inflor
inflor_shoot
meristem_Wus
meristem_Clavata3
meristem_Fil
young_leaf
mature_leaf
seedling
cotyledon
whole_plant
senescent_leaf
shoot
cauline_leaf
internode_shoot
hypocotyl
early_rosette
pedicel
leaf
root_d17
root_endodermis
root_stele
root_xylem
root_d7
root_cortex
root_epidermis
root_protophloem
lateral_root_cap
root_pericycle
root_companion_cell
silique_glob_emb
silique_heart_emb
seed_torpedo
seed_early_green_cot
seed_coat_globEmb
micropylar_endosperm_globEmb
embryo_proper_globEmb
chalazal_endosperm_globEmb

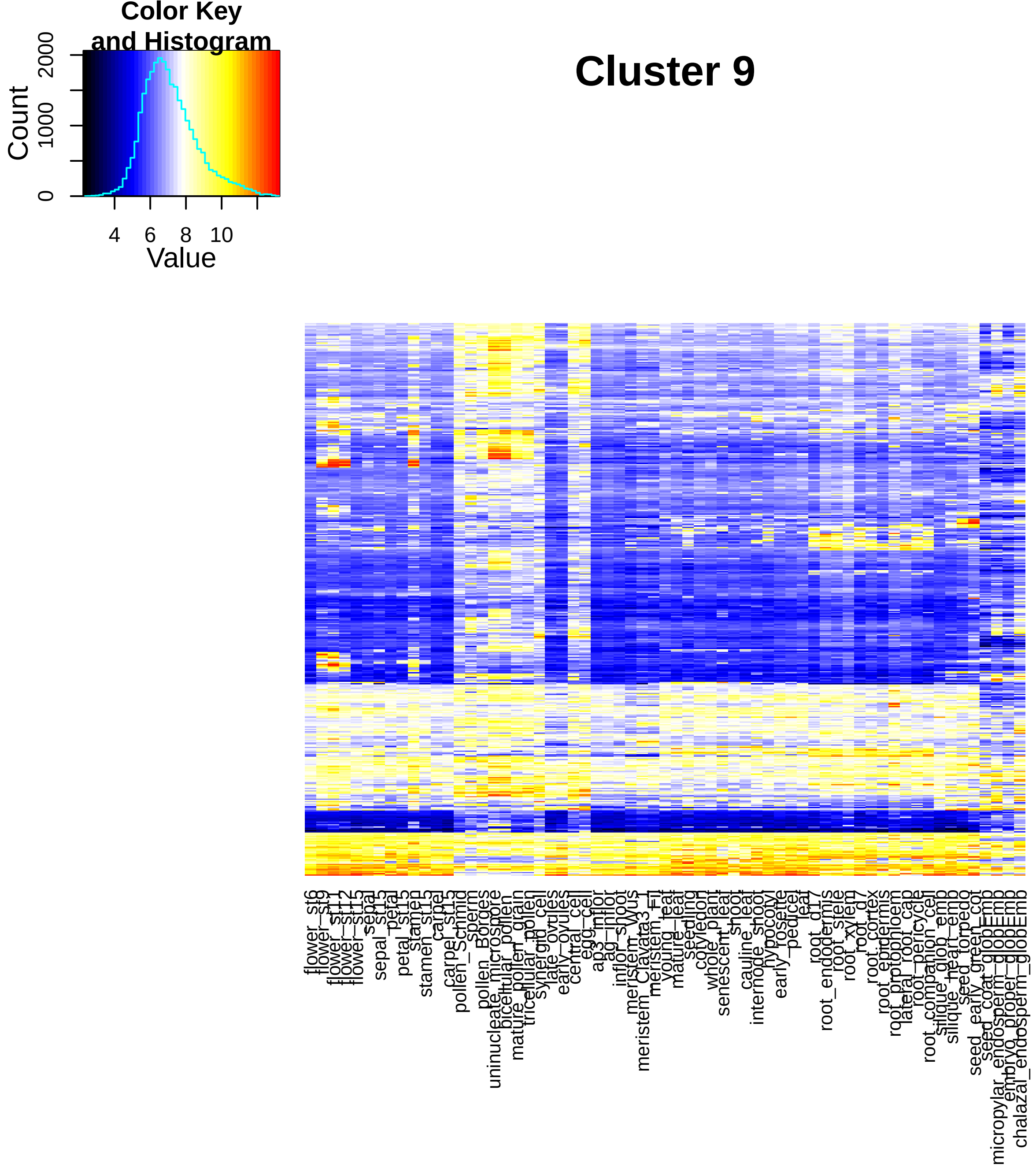

Color Key and Histogram
Count
0
1000
2000
4
6
8
10
Value
Cluster 9
flower_st6
flower_st9
flower_st11
flower_st12
flower_st15
sepal
sepal_st15
petal
petal_st15
stamen
stamen_st15
carpel
carpel_st15
pollen_Schmid
sperm
pollen_Borges
uninucleate_microspore
bicellular_pollen
mature_pollen_grain
tricellular_pollen
synergid_cell
late_ovules
early_ovules
central_cell
egg_cell
ap3_inflor
ag_inflor
inflor_shoot
meristem_Wus
meristem_Clavata3
meristem_Fil
young_leaf
mature_leaf
seedling
cotyledon
whole_plant
senescent_leaf
shoot
cauline_leaf
internode_shoot
hypocotyl
early_rosette
pedicel
leaf
root_d17
root_endodermis
root_stele
root_xylem
root_d7
root_cortex
root_epidermis
root_protophloem
lateral_root_cap
root_pericycle
root_companion_cell
silique_glob_emb
silique_heart_emb
seed_torpedo
seed_early_green_cot
seed_coat_globEmb
micropylar_endosperm_globEmb
embryo_proper_globEmb
chalazal_endosperm_globEmb

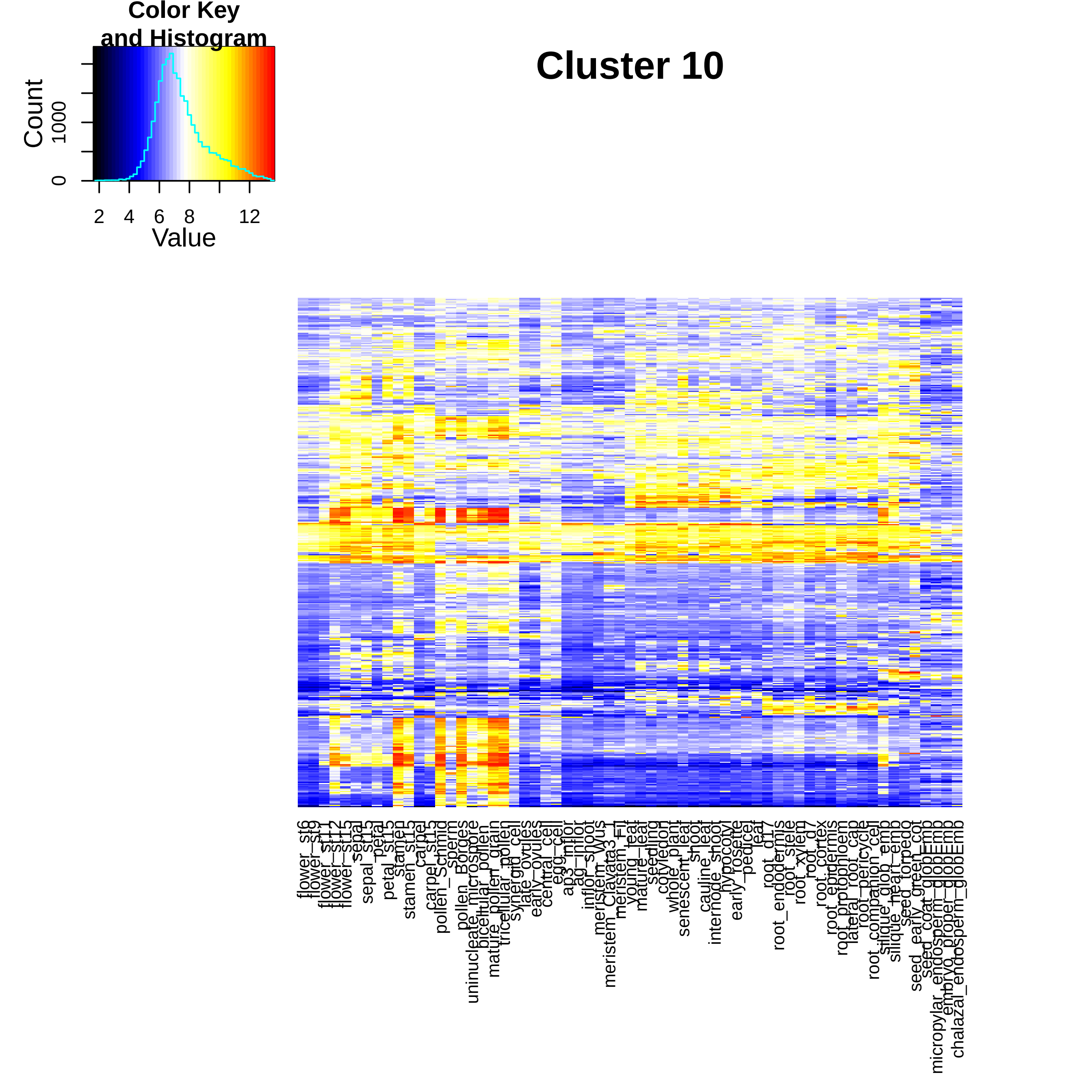

Color Key
and Histogram
Count
0
1000
2
4
6
8
12
Value
Cluster 10
flower_st6
flower_st9
flower_st11
flower_st12
flower_st15
sepal
sepal_st15
petal
petal_st15
stamen
stamen_st15
carpel
carpel_st15
pollen_Schmid
sperm
pollen_Borges
uninucleate_microspore
bicellular_pollen
mature_pollen_grain
tricellular_pollen
synergid_cell
late_ovules
early_ovules
central_cell
egg_cell
ap3_inflor
ag_inflor
inflor_shoot
meristem_Wus
meristem_Clavata3
meristem_Fil
young_leaf
mature_leaf
seedling
cotyledon
whole_plant
senescent_leaf
shoot
cauline_leaf
internode_shoot
hypocoty
early_rosette
pedicel
leaf
root_d17
root_endodermis
root_stele
root_xylem
root_d7
root_cortex
root_epidermis
root_protophloem
lateral_root_cap
root_pericycle
root_companion_cell
silique_glob_emb
silique_heart_emb
seed_torpedo
seed_early_green_cot
seed_coat_globEmb
micropylar_endosperm_globEmb
embryo_proper_globEmb
chalazal_endosperm_globEmb

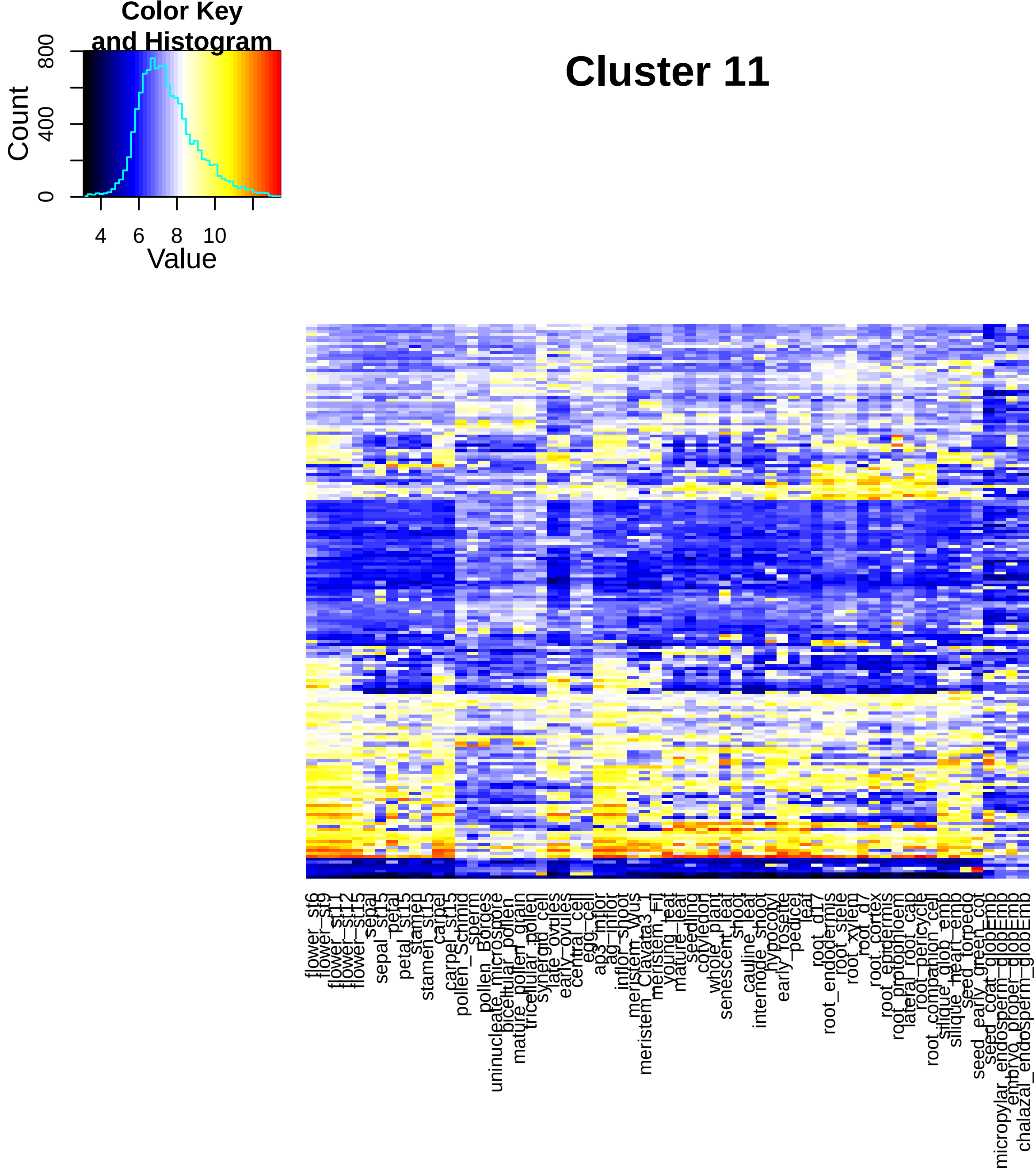

Color Key
and Histogram
Count
Value
0
400
800
4
6
8
10
Cluster 11
flower_st6
flower_st9
flower_st11
flower_st12
flower_st15
sepal
sepal_st15
petal
petal_st15
stamen
stamen_st15
carpel
carpel_st15
pollen_Schmid
sperm
pollen_Borges
uninucleate_microspore
bicellular_pollen
mature_pollen_grain
tricellular_pollen
synergid_cell
late_ovules
early_ovules
central_cell
egg_cell
ap3_inflor
ag_inflor
inflor_shoot
meristem_Wus
meristem_Clavata3
meristem_Fil
young_leaf
mature_leaf
seedling
cotyledon
whole_plant
senescent_leaf
shoot
cauline_leaf
internode_shoot
hypocotyl
early_rosette
pedicel
leaf
root_d17
root_endodermis
root_stele
root_xylem
root_d7
root_cortex
root_epidermis
root_protophloem
lateral_root_cap
root_pericycle
root_companion_cell
silique_glob_emb
silique_heart_emb
seed_torpedo
seed_early_green_cot
seed_coat_globEmb
micropylar_endosperm_globEmb
embryo_proper_globEmb
chalazal_endosperm_globEmb

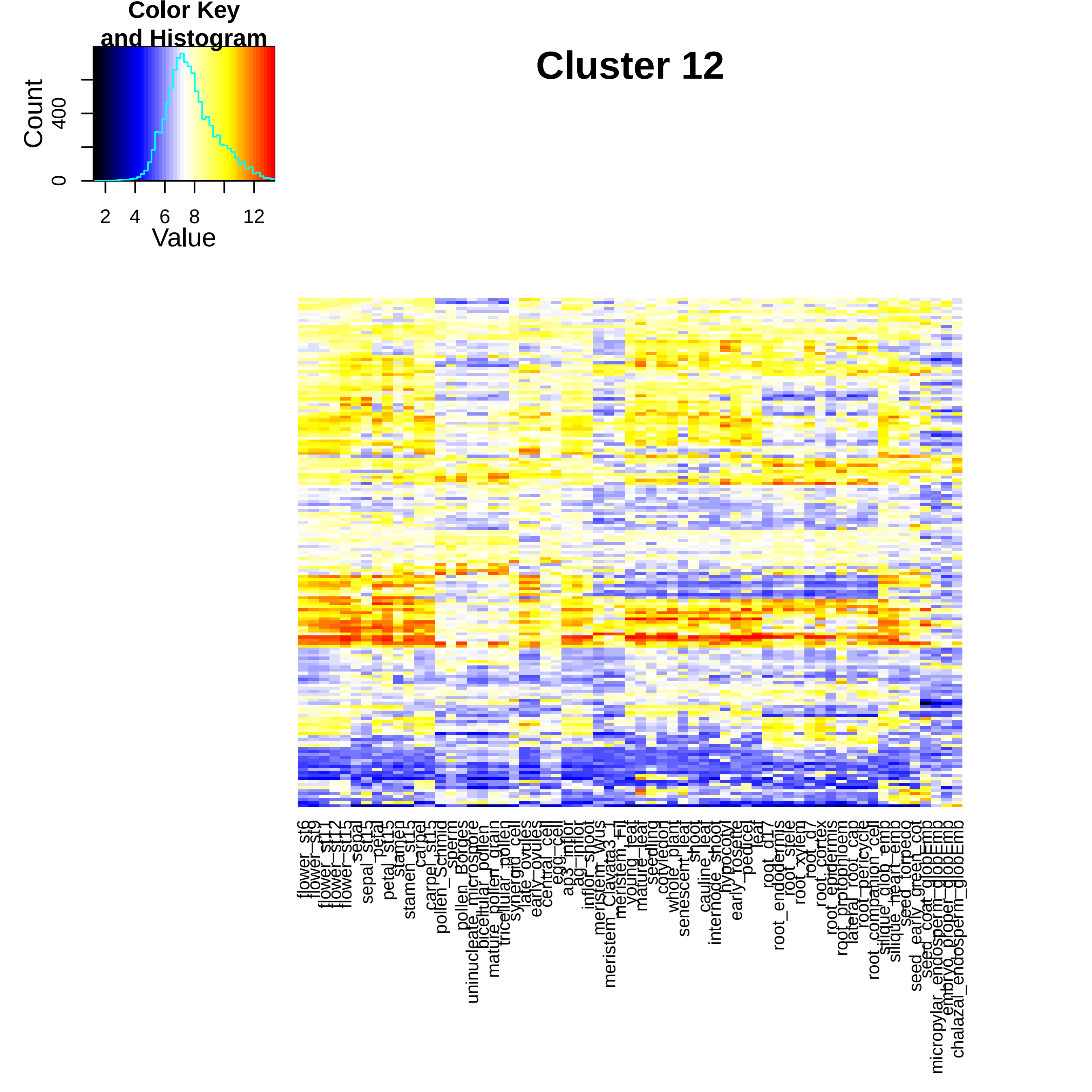
Color Key
and Histogram
Count
0
400
2
4
6
8
12
Value
Cluster 12
flower_st6
flower_st9
flower_st11
flower_st12
flower_st15
sepal
sepal_st15
petal
petal_st15
stamen
stamen_st15
carpel
carpel_st15
pollen_Schmid
sperm
pollen_Borges
uninucleate_microspore
bicellular_pollen
mature_pollen_grain
tricellular_pollen
synergid_cell
late_ovules
early_ovules
central_cell
egg_cell
ap3_inflor
ag_inflor
inflor_shoot
meristem_Wus
meristem_Clavata3
meristem_Fil
young_leaf
mature_leaf
seedling
cotyledon
whole_plant
senescent_leaf
shoot
cauline_leaf
internode_shoot
hypocotyl
early_rosette
pedicel
leaf
root_d17
root_endodermis
root_stele
root_xylem
root_d7
root_cortex
root_epidermis
root_protophloem
lateral_root_cap
root_pericycle
root_companion_cell
silique_glob_emb
silique_heart_emb
seed_torpedo
seed_early_green_cot
seed_coat_globEmb
micropylar_endosperm_globEmb
embryo_proper_globEmb
chalazal_endosperm_globEmb

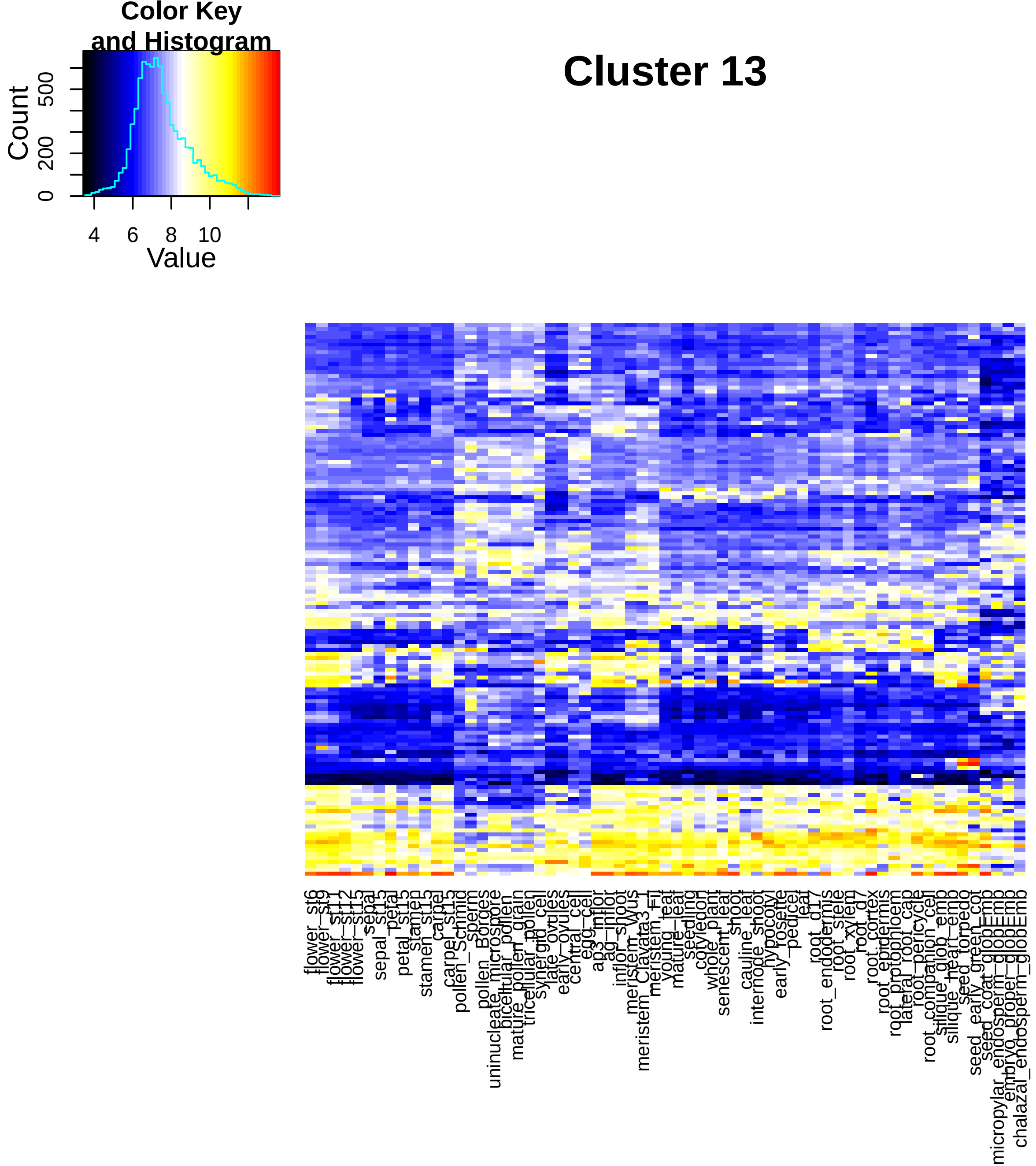

Color Key
and Histogram
Count
0
200
500
4
6
8
10
Value
Cluster 13
flower_st6
flower_st9
flower_st11
flower_st12
flower_st15
sepal
sepal_st15
petal
petal_st15
stamen
stamen_st15
carpel
carpel_st15
pollen_Schmid
sperm
pollen_Borges
uninucleate_microspore
bicellular_pollen
mature_pollen_grain
tricellular_pollen
synergid_cell
late_ovules
early_ovules
central_cell
egg_cell
ap3_inflor
ag_inflor
inflor_shoot
meristem_Wus
meristem_Clavata3
meristem_Fil
young_leaf
mature_leaf
seedling
cotyledon
whole_plant
senescent_leaf
shoot
cauline_leaf
internode_shoot
hypocoty
early_rosette
pedicel
leaf
root_d17
root_endodermis
root_stele
root_xylem
root_d7
root_cortex
root_epidermis
root_protophloem
lateral_root_cap
root_pericycle
root_companion_cell
silique_glob_emb
silique_heart_emb
seed_torpedo
seed_early_green_cot
seed_coat_globEmb
micropylar_endosperm_globEmb
embryo_proper_globEmb
chalazal_endosperm_globEmb

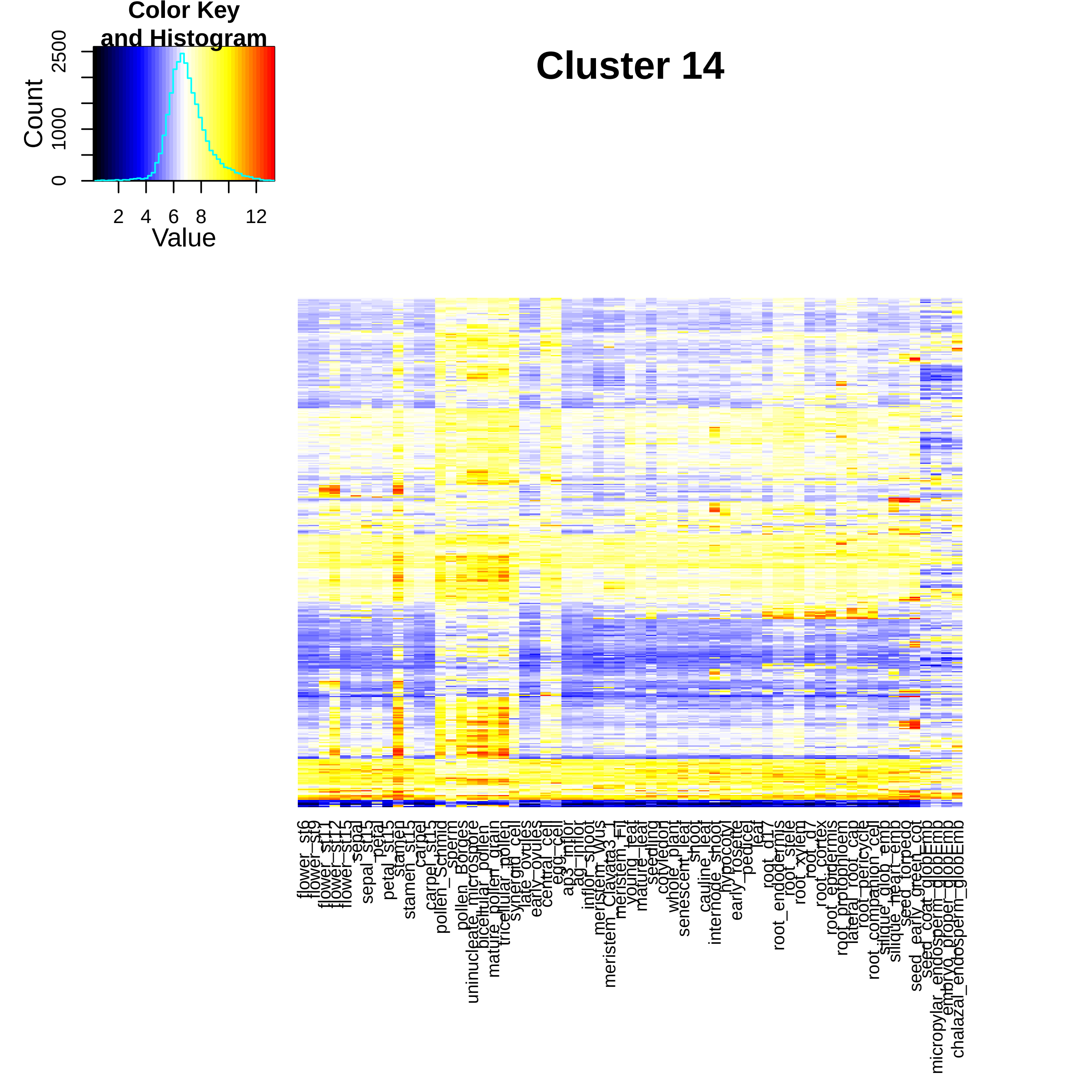
Color Key
and Histogram
Count
0
1000
2500
2
4
6
8
12
Value
Cluster 14
flower_st6
flower_st9
flower_st11
flower_st12
flower_st15
sepal
sepal_st15
petal
petal_st15
stamen
stamen_st15
carpel
carpel_st15
pollen_Schmid
sperm
pollen_Borges
uninucleate_microspore
bicellular_pollen
mature_pollen_grain
tricellular_pollen
synergid_cell
late_ovules
early_ovules
central_cell
egg_cell
ap3_inflor
ag_inflor
inflor_shoot
meristem_Wus
meristem_Clavata3
meristem_Fil
young_leaf
mature_leaf
seedling
cotyledon
whole_plant
senescent_leaf
shoot
cauline_leaf
internode_shoot
hypocotyl
early_rosette
pedicel
leaf
root_d17
root_endodermis
root_stele
root_xylem
root_d7
root_cortex
root_epidermis
root_protophloem
lateral_root_cap
root_pericycle
root_companion_cell
silique_glob_emb
silique_heart_emb
seed_torpedo
seed_early_green_cot
seed_coat_globEmb
micropylar_endosperm_globEmb
embryo_proper_globEmb
chalazal_endosperm_globEmb

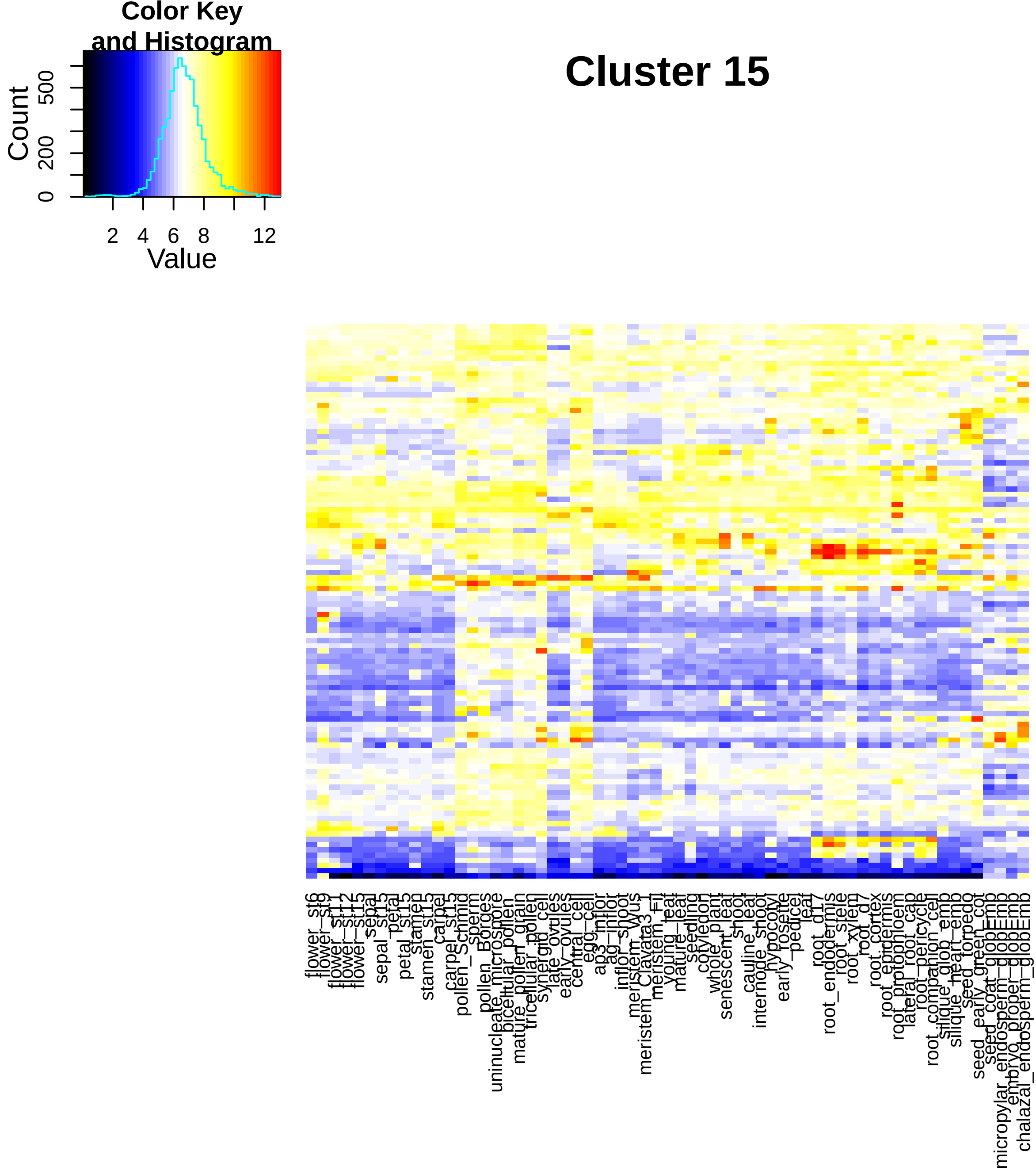
Color Key
and Histogram
Count
0
200
500
2
4
6
8
12
Value
Cluster 15
flower_st6
flower_st9
flower_st11
flower_st12
flower_st15
sepal
sepal_st15
petal
petal_st15
stamen
stamen_st15
carpel
carpel_st15
pollen_Schmid
sperm
pollen_Borges
uninucleate_microspore
bicellular_pollen
mature_pollen_grain
tricellular_pollen
synergid_cell
late_ovules
early_ovules
central_cell
egg_cell
ap3_inflor
ag_inflor
inflor_shoot
meristem_Wus
meristem_Clavata3
meristem_Fil
young_leaf
mature_leaf
seedling
cotyledon
whole_plant
senescent_leaf
shoot
cauline_leaf
internode_shoot
hypocotyl
early_rosette
pedicel
leaf
root_d17
root_endodermis
root_stele
root_xylem
root_d7
root_cortex
root_epidermis
root_protophloem
lateral_root_cap
root_pericycle
root_companion_cell
silique_glob_emb
silique_heart_emb
seed_torpedo
seed_early_green_cot
seed_coat_globEmb
micropylar_endosperm_globEmb
embryo_proper_globEmb
chalazal_endosperm_globEmb
